# Lamin A/C impairments cause mitochondrial dysfunction by attenuating PGC1α and the NAMPT-NAD^+^ pathway

**DOI:** 10.1093/nar/gkac741

**Published:** 2022-09-13

**Authors:** Scott Maynard, Arnaldur Hall, Panagiotis Galanos, Salvatore Rizza, Tatsuro Yamamoto, Helena Hagner Gram, Sebastian H N Munk, Muhammad Shoaib, Claus Storgaard Sørensen, Vilhelm A Bohr, Mads Lerdrup, Apolinar Maya-Mendoza, Jiri Bartek

**Affiliations:** Danish Cancer Society Research Center, DK-2100 Copenhagen, Denmark; Danish Cancer Society Research Center, DK-2100 Copenhagen, Denmark; Danish Cancer Society Research Center, DK-2100 Copenhagen, Denmark; Danish Cancer Society Research Center, DK-2100 Copenhagen, Denmark; Danish Cancer Society Research Center, DK-2100 Copenhagen, Denmark; Danish Cancer Society Research Center, DK-2100 Copenhagen, Denmark; Danish Cancer Society Research Center, DK-2100 Copenhagen, Denmark; Biotech Research and Innovation Centre (BRIC), University of Copenhagen, DK-2200 Copenhagen, Denmark; Biotech Research and Innovation Centre (BRIC), University of Copenhagen, DK-2200 Copenhagen, Denmark; Department of Cellular and Molecular Medicine, Center for Healthy Aging, University of Copenhagen, DK-2200 Copenhagen, Denmark; Laboratory of Molecular Gerontology, National Institute on Aging, National Institutes of Health, Baltimore, MD 21224, USA; The DNRF Center for Chromosome Stability, Department of Cellular and Molecular Medicine, University of Copenhagen, DK-2200 Copenhagen, Denmark; Danish Cancer Society Research Center, DK-2100 Copenhagen, Denmark; Danish Cancer Society Research Center, DK-2100 Copenhagen, Denmark; Division of Genome Biology, Department of Medical Biochemistry and Biophysics, Science for Life Laboratory, Karolinska Institute, SE-17177 Stockholm, Sweden

## Abstract

Mutations in the lamin A/C gene (*LMNA*) cause laminopathies such as the premature aging Hutchinson Gilford progeria syndrome (HGPS) and altered lamin A/C levels are found in diverse malignancies. The underlying lamin-associated mechanisms remain poorly understood. Here we report that lamin A/C-null mouse embryo fibroblasts (*Lmna*^−/−^ MEFs) and human progerin-expressing HGPS fibroblasts both display reduced NAD^+^ levels, unstable mitochondrial DNA and attenuated bioenergetics. This mitochondrial dysfunction is associated with reduced chromatin recruitment (*Lmna*^−/−^ MEFs) or low levels (HGPS) of PGC1α, the key transcription factor for mitochondrial homeostasis. *Lmna*^−/−^ MEFs showed reduced expression of the NAD^+^-biosynthesis enzyme NAMPT and attenuated activity of the NAD^+^-dependent deacetylase SIRT1. We find high PARylation in lamin A/C-aberrant cells, further decreasing the NAD^+^ pool and consistent with impaired DNA base excision repair in both cell models, a condition that fuels DNA damage-induced PARylation under oxidative stress. Further, ATAC-sequencing revealed a substantially altered chromatin landscape in *Lmna*^−/−^ MEFs, including aberrantly reduced accessibility at the *Nampt* gene promoter. Thus, we identified a new role of lamin A/C as a key modulator of mitochondrial function through impairments of PGC1α and the NAMPT-NAD^+^ pathway, with broader implications for the aging process.

## INTRODUCTION

The nuclear lamina is a fibrillar network, formed by lamins (A- and B-type) and lamin-associated proteins, that lies at the inner periphery of the nucleus and interacts with the chromatin and various proteins in the nucleus (1,2). In somatic cells of humans, mice, and most vertebrates, the major isoforms of the A-type lamins are lamin A and C (lamin A/C), which arise from the lamin A gene (*LMNA*) by alternative splicing. A- and B- type lamins are type V intermediate filament proteins that have important scaffolding roles in the nucleus, providing mechanical support and regulating cellular processes such as gene expression, cell differentiation, and the DNA damage response (3–5). Importantly, the nuclear lamina associates with many histone deacetylases, helping to preserve their activity, thus regulating chromatin structure and gene expression (6,7). Many cancers show altered levels of lamin A/C (8) and accumulating evidence implicates lamin aberrations in carcinogenesis (4,9) and altered cancer cell migration, a key feature of invasiveness and metastasis (10).

Mutations in *LMNA* have been linked to physiological aging, as well as degenerative disorders, broadly termed laminopathies, including muscular dystrophy, neuropathies, lipodystrophies, and the premature aging syndrome Hutchinson-Gilford Progeria syndrome (HGPS) (3,11–16). HGPS is a rare genetic disorder characterized by early onset of symptoms resembling normal aging. Most HGPS patients die before the age of 20, primarily due to cardiovascular problems and heart failure. Notably, there is an absence of neuronal degeneration, and a rarity of cancer, during the short lifespan of the HGPS patients. Most HGPS cases carry the *LMNA* G608G (GGC > GGT) mutation within exon 11 of *LMNA*. This mutation activates a splice donor site that results in production of a dominant negative form of lamin A, called progerin. The aberrant splicing event that leads to progerin expression also occurs, at much lower levels, in normal aging (17). At the cellular level, progerin forms insoluble toxic aggregates. The accumulation of this mutant protein leads to a dysfunctional nuclear membrane, premature cell senescence, apoptosis, dysregulation of gene expression, and accumulation of DNA damage (15,18). These defects appear to contribute to the clinical features of premature aging in HGPS patients.

Nuclear and mitochondrial functions are tightly integrated through several levels of signaling (19,20) and accumulating evidence suggests that proper levels of nuclear lamins may be important in mitochondrial respiration. For example, it has been shown that lamin A/C depletion in human fibroblasts leads to oxidative stress and impaired basal mitochondrial respiration (21), and that adipose tissue from mice that do not express the lamin A isoform (lamin C-only mice) have a reduced number of mitochondria and attenuated mitochondrial bioenergetics (22). There are also reports showing the effects of progerin on mitochondrial respiration. However, the results from these studies are inconsistent. It has been reported that basal mitochondrial respiration is enhanced in progerin-expressing 3T3L1 murine preadipocytes (23), and that mitochondrial mass and respiration are enhanced in cells from an HGPS mouse model (22). However, it has also been reported that human HGPS fibroblasts display decreased levels of electron transport chain (ETC) components (24), and mitochondrial respiration defects (21,25).

Research has shown that NAD^+^ acts as a signaling molecule in cellular metabolism, including regulation of mitochondrial respiration (26). Reduced levels of NAD^+^ and NAMPT (rate limiting enzyme in the salvage pathway for NAD^+^ biosynthesis) are found in multiple organs with age (27). Also, aging-associated inflammation and oxidative stress have been shown to result in reduced NAMPT-mediated NAD^+^ biosynthesis (27). NAD^+^ is a rate-limiting metabolite for PARP1 activation. PARP1 metabolizes NAD^+^ to give rise to PAR chains (PARylation) on target proteins, including PARP1 itself. PARylation is induced by DNA alterations, including breaks generated from oxygen species (28). PARylation of PARP1 and other proteins is important in the regulation of DNA repair (29) and is associated with carcinogenesis (30). NAD^+^ is also a substrate for the deacetylase activity of the sirtuin proteins (31). Regulation of the level or activity of sirtuins is important in maintaining optimal metabolic homeostasis through a variety deacetylation targets (31–33). SIRT1 is activated in times of energy demand by high levels of NAD^+^. The NAD^+^-dependent SIRT1-PGC1α axis regulates mitochondrial biogenesis (34). Dysfunction in this pathway can play a role in diseases associated with aging (35–37). There is recent evidence suggesting that PGC1α may promote de novo NAD^+^ biosynthesis under oxidative stress (38,39). The reduction in NAD^+^ levels during DNA damage-induced PARylation and other NAD^+^ consuming processes, or by impaired NAD^+^ biosynthesis, can reduce NAD^+^ activation of sirtuins. Since nuclear NAD^+^-dependent enzymes regulate mitochondrial function, and lamins are nuclear scaffolding proteins, we hypothesized that lamins may regulate mitochondrial respiration through NAD^+^ signaling.

Here, we show that two mammalian cell models, each with a distinct lamin A/C aberration, namely *Lmna*^−/−^ mouse embryo fibroblasts (MEFs) and progerin-expressing human HGPS dermal fibroblasts, share reduced levels of NAD^+^, impairment of PGC1α capability and mitochondrial dysfunction. Given the multifaceted roles of NAD^+^ and PGC1α in regulating cellular and metabolic signaling, including the involvement of NAD^+^ signaling in genome integrity maintenance, our findings described in detail below have broad implications for cellular pathophysiology including premature aging and aging-associated pathologies.

## MATERIALS AND METHODS

### Cell culture and treatments

MEFs (spontaneously immortalized) and HGPS dermal fibroblasts were grown in Dulbecco modified eagle medium (DMEM), supplemented with 10% fetal bovine serum (FBS) (Gibco BRL), 50 μg/ml streptomycin and 50 units/ml penicillin, at 37°C, 5% CO_2_. MEFs were incubated under 20% oxygen. All HGPS fibroblasts and normal counterpart fibroblasts were incubated under 3% oxygen to delay the onset of oxygen-induced senescence in the HGPS cells and to mimic physiological conditions. The *Lmna*^−/−^ and *Lmna*^+/+^ MEFs were generated in the laboratory of Colin L. Stewart (40). Fibroblasts from HGPS patients and normal counterparts (parents) were purchased from Coriell Institute. The three Normal/HGPS pairs used in this study have the following catalogue numbers with indicated patient age: Normal-1/HGPS-1 = AG03258 (36 y/o father)/AG03198 (10 y/o female), Normal-2/HGPS-2 = AG03512 (41 y/o mother)/AG03513 (13 y/o male) and Normal-3 /HGPS-3 = AG06299 (34 y/o mother)/AG06917 (3 y/o male). To inhibit PARylation, the PARP1 inhibitor olaparib (2 μM) was added to the medium of cultured MEFs, and incubation carried out for 2 h. To activate PARylation, 2 mM NAD^+^ was added to the medium of cultured MEFs, and incubation carried out for 24 h. To test for the effect of oxidative stress on SIRT1 activation (deacetylation of p53 visualized on western blot), MEFs were treated with 800 μM of H_2_O_2_ for 5 h. The SIRT1 inhibitor (selisistat, also called EX52; Selleckchem) was added to cultured MEFs at 2 μM for 24 h.

### Microarray

The DNA microarray data reported for the *Lmna*^−/−^ MEFs (relative to *Lmna*^+/+^ MEFs) was mined from our recent publication (4). The DNA microarray experiments and analysis on HGPS fibroblasts and normal fibroblasts (three pairs, which are listed above) were performed as described previously (4). Raw hybridization intensity data were log-transformed and normalized to yield *z*-scores, as an indication of the extent of elevated or inhibited (negative value) gene expression, as a ranking of gene ontology terms. The experiments and analysis were performed at the microarray facility of the National Institute on Aging, Intramural Research Program, National Institute of Health (NIH).

### Western blotting and chromatin enriched fractionation

Proteins were separated on 12% Tris-glycine gels (Bio-Rad) in Novex tris-glycine SDS running buffer (Life Technologies). Whole cell extracts were prepared in lysis buffer containing 50 mM Tris–HCl, 150 mM NaCl, 1 mM EDTA, 1% triton X-100, with 1× mini EDTA-free protease inhibitor cocktail (Sigma) and 1× PhosSTOP phosphatase inhibitor (Sigma). In the case of extracting chromatin bound enriched fraction, ice-cold TNE buffer containing 0.1% NP40 was added, and cells were incubated for 10 min on ice. Then, cells were rinsed three times with PBS before proceeding to protein extraction as followed with whole cell extracts. Tris-glycine SDS sample buffer was then added for loading. Transfer to PVDF membranes (Life Technologies) was carried out by electroblotting in Novex tris-glycine transfer buffer (Life Technologies) containing 15% methanol, for 1 h at 100 V. Membranes were blocked 1 h at room temperature in 5% nonfat dry milk in TBST (20 mM Tris–HCl pH 7.2, 137 mM NaCl, 0.1% Tween-20). All antibodies were diluted in 3% milk in TBST. Secondary HRP-conjugated antibodies (Jackson ImmunoResearch) and Pierce ECL Plus (ThermoScientific) were used to visualize the protein bands on high performance chemiluminescence film (GE Healthcare). The films were scanned and saved as 16-bit grayscale tiff files.

### Antibodies

For western blotting: β-actin (AMB-7229; Nordic Biosite), α-tubulin (ab4074, Abcam), Histone H3 (ab1791, Abcam), lamin A/C (sc-20681; Santa Cruz), progerin (ab66587; Abcam), PAR (ab14460; Abcam), SIRT1 (04-1557; Millipore), PGC1α (NBP1-04676ss; Novus), NAMPT (A0256; ABclonal), p53 (sc-126; Santa Cruz), p53-acetyl K382 (ab75754; Abcam), Total OXPHOS (ab110413, Abcam). For immunofluorescence: TOM20 (ab56783; Abcam), dsDNA (ab27156, Abcam), LC3 (ab192890; Abcam).

### Analyses of mitochondrial mass, mitochondrial transmembrane potential and ROS

Total mitochondrial mass and mitochondrial transmembrane potential (Δψ_m_) were analyzed by incubating cells with 50 nM MitoTracker Green FM or 200 nM TMRM (Thermo-Fisher Scientific), respectively, in DMEM without serum and phenol red (Thermo-Fisher Scientific) for 30 min. Stained cells were washed twice with cold PBS, collected and analyzed by flow cytometry (FACS Verse, BD Biosciences). Normalized Δψ_m_ was calculated as TMRM/MitoTracker Green FM relative fluorescence (geometric mean). Reactive oxygen species (ROS) were analyzed by incubating cells for 15 min in serum-free DMEM with 5 μM of the superoxide indicator dihydroethidium (DHE, Thermo-Fisher Scientific), or, alternatively, with 5 μM of 2',7'-dichlorodihydrofluorescein diacetate (H_2_DCFDA, Thermo-Fisher Scientific), a chemically reduced form of fluorescein used as a general indicator for ROS. Stained cells were washed twice with cold PBS, collected and analyzed by flow cytometry (FACS Verse, BD-Biosciences). For both probes, fluorescence intensity (geometric mean) was recorded (DHE, Ex/Em: 510/580 nm; H_2_DCFDA Ex/Em: 495/517 nm) and values were expressed as relative fold change with respect to normal cells.

### Quantitative RT-PCR

Total RNA samples were isolated using RNeasy Mini Kit (Qiagen Sciences) according to the manufacturer's protocol. First strand cDNA was synthesized from 2 μg of total RNA with random hexamer primers using High-Capacity cDNA Reverse Transcription kit (Applied Biosystems). qRT-PCR was performed using the ABI Prism 7300 system (Applied Biosystems) and SYBR Select Master Mix containing SYBR Green dye (Applied Biosystems). The relative quantity of cDNA was estimated by the DDCT method and data were normalized to β-actin. The primers were purchased from Eurofins Genomics. The primers sequences used for the mouse *Nampt* gene are as follows. Forward: 5′-ACAGATACTGTGGCGGGATTG-3′ and reverse: 5′-TGATATCCACGCCATCTCCTTG-3′.

### Mitochondrial mass and mtDNA analysis by quantitative image-based cytometry (QIBC)

Immunofluorescence was performed on MEFs and HGPS cells fixed with 4% paraformaldehyde at room temperature for 30 min, and incubated with antibodies for TOM20 to detect mitochondria, or for double stranded DNA (dsDNA) to detect mtDNA. The slides were mounted with DAPI-containing Vectashield (Vector laboratories). Scanning confocal microscope LSM800 (Zeiss) was used to acquire the images in 63× magnification. Acquired images were then processed by Blue ZEN software (Zeiss). Quantitative image-based cytometry (QIBC) was performed as previously described (41). Non-overlapping images were acquired in an unbiased and automated fashion with ScanR acquisition software and the Olympus ScanR microscope. Acquisition time was adjusted for each channel to avoid saturation. For each individual condition, at least 243 images were acquired, divided equally in 3 independent coverslips/experiments with each containing at least 1500 cells. Automated focus was performed using the DAPI channel. Hardware autofocus, Coarse and Fine Software autofocus were performed diagonally and in total 17 out of 81 images for ideal calibration, while for the remaining images Coarse and Fine was used. Images were acquired with an Olympus UPLSAPO 40×. Automated image analysis was performed with ScanR image analysis software and the results were exported as .txt. files and subjected to further analysis with Spotfire analysis program.

### Comet assay

The FPG-comet assay (single cell gel electrophoresis assay) was performed as described previously (4). The cells were treated with 100 μM H_2_O_2_ for 1h, then the medium replaced with H_2_O_2_-free medium, and cells collected at the indicated time points for comet assay. In order to specifically determine the extent of oxidative DNA damage, the slides were treated with 5 U of formamidopyrimidine DNA glycosylase (FPG) (4040-500-01; Trevigen, 5 U/μl) per slide [1 μl FPG in 99 μl FLARE buffer I (Trevigen) containing 0.1 mg/ml BSA] and incubated for 1 h at 37°C; corresponding slides with buffer/BSA-only (‘no-FPG’) were included as controls. FPG is specific for oxidized purines, including 8-oxo-7,8-dihydroguanine (8-oxoGua), 2,6-diamino-4-hydroxy-5-formamidopyrimidine (FaPyGua) and 4,6-diamino-5-formamidopyrimidine (FaPyAde) and other ring-opened purines (42). Average of FPG-sensitive sites (estimate for the extent of oxidative lesions) was calculated as average comet tail intensity of FPG treated cells minus average comet tail intensity of buffer/BSA treated cells. DNA repair efficiency was expressed as percent of FPG-sensitive sites remaining, at the indicated repair times, relative to 5 min of repair, after correction for the FPG-sensitive sites in untreated cells (*n* = 100 comet tails).

### NAD^+^ quantitation

Determination of cellular NAD^+^ levels was performed using the NAD/NADH Assay Kit (Colorimetric) (ab65348; Abcam) according to the manufacturer's instructions. NAD^+^ was calculated as Total NAD minus NADH. Values obtained were corrected for protein concentration of the extract or cell numbers.

### Assay for transposase-accessible chromatin using sequencing (ATAC-seq)

ATAC-seq was performed as described by Buenrostro et al. (43) with a few modifications. Briefly, 50 000 cells were washed with 50 μl ice-cold 1× PBS followed by centrifugation at 500× g for 5 min. Cells were lysed using cold lysis buffer (10 mM Tris–HCl pH 7.5, 10 mM NaCl, 3 mM MgCl_2_, 0.1% NP40, 0.1% Tween-20 and 0.01% Digitonin) and gently pipetted up and down 5 times followed by 3 min incubation on ice. 500 μl of Wash buffer (10 mM Tris–HCl pH 7.5, 10 mM NaCl, 3 mM MgCl_2_ and 0.1% Tween-20) was added, followed by centrifugation at 500 × g for 10 min using a refrigerated centrifuge to collect nuclei. The nuclei pellet was resuspended in the transposase reaction mix containing 25 μl of 2× TD buffer, 2.5 μl transposase (Illumina, FC-121-1031), 0.1% Tween-20 and 0.01% Digitonin and nuclease-free water up to 50 μl). The nuclei were resuspended by gently pipetting up and down 6–8 times and incubated at 37°C for 30 min on thermomixer at 1000 rpm. DNA was then purified using a Qiagen MinElute Kit (Cat No./ID: 28004) in 10 μl of elution buffer. After purification, the DNA fragments were amplified using Nextera PCR master mix (NPM) and 1.25 μM of Nextera PCR Index primers 1 and 2 (Illumina; FC-121–1011), using the following PCR conditions: 72°C for 5 min; 98°C for 30 s; and 5 cycles of 98°C for 10 s, 63°C for 30 s, and 72°C for 1 min. We performed the size selection (<600 bp) using Ampure XP magnetic beads (Beckman Coulter Inc.) according to manufacturer's protocol. To reduce GC and size bias in our PCR, we performed a quantitative real-time PCR (qPCR)-based library quantification. First, one fifth of the purified PCR product was amplified using 2× KAPA SYBR FAST qPCR Master mix (KK4932) for 20 cycles. The optimal number of cycles were determined by the cycle number that corresponds to one fourth of maximum fluorescent intensity (usually around 6–7 cycles). The full libraries were then amplified for the corresponding number of cycles (determined in previous step) for each sample. The libraries were again purified with size selection (<600 bp) using Ampure XP magnetic beads according to the manufacturer's protocol. Libraries were quantified using the Qubit DNA HS Kit, and for quality control, 1 μl (around 5 ng) of each sample was run on Bioanalyzer High Sensitivity DNA Chip. In all, 2 nM of all libraries were pooled and 1.5 pM were sequenced on Illumina NextSeq500 (500/550 High Output v2 Kit—150 cycles) having a read length of 2 × 76 bp (paired-end).

#### ATAC-seq data preprocessing

The quality of sequenced reads was analyzed using FastQC (v. 0.10.1) (44) and FastqScreen (v. 0.11.4) (45), and summarized using MultiQC (v. 1.7) (46). ATAC-seq data were processed and mapped as previously described (47). Briefly, the raw paired-end reads were first trimmed for Nextera transposase adapter sequences using Trimmomatic (v0.32) (48) in palindrome mode with default settings except ILLUMINACLIP:2:30:10:1:true MINLEN:25. FastQC of reads before and after trimming confirmed the removal of any 3′ adapter sequences, while also clearly showing the known insertion Tn5 motif in the 5′-ends. The trimmed PE reads were mapped to the hg19 assembly (canonical chromosomes only) using bowtie2 v.2.2.9 (49) with default settings except -k 2 -X 2000 –no-mixed –nodiscordant. After sorting (SortSam) and labeling duplicates (MarkDuplicates) with Picard tools (v. 2.6.0–27) (https://broadinstitute.github.io/picard) and adding a NH tag (number of reported alignments), reads were filtered to exclude unmapped, multimapping, and mitochondrial reads (samtools view -f 2 -F 4 (50) and custom filter). The filtered bam files were converted to bed format using bedtools bamtobed (v2.26.0-92) (51), and read start and stop coordinates were finally adjusted by +5 bp and −4 bp, respectively, to adjust for Tn5-binding properties as previously described (52).

#### ATAC-seq data analysis and integration

Mapped reads were deduplicated and imported into EaSeq v. 1.2 (53) using default settings. ATAC-seq peaks were called using MACS2 (54), and imported into EaSeq as regionsets. Gene annotations were imported as ‘Geneset’ from RefSeq (55) using the integrated tool. LADs and enhancer (downloaded from http://chromosome.sdsc.edu/mouse/download.html) coordinates in MEFs were previously published (56,57) and converted to mm10 coordinates using the liftover tool at UCSC (58). Loci of interest as well as expression data were imported into EaSeq as ‘Regionsets’, and when nothing else is mentioned subsequent visualization was done using EaSeq and integrated tools as previously described (59). Imported peaks were all merged using the ‘Modify Regionset’-tool in EaSeq and the ‘Merge with’ option successively until all peaksets were merged. The number of fragments per kilobase pair per million reads (FPKM) was quantified in the +/-500bp area surrounding each peak center using the ‘Quantify’-tool in EaSeq with default settings. To minimize global variation in ATAC-seq probe integration efficiencies, quantified values were further quantile normalized using the ‘normalize’-tool in EaSeq with default settings, and only peaks with an average value in all replicates of 1 or more were used for subsequent analyses and visualization. For differential enrichment analysis, unnormalized read counts were obtained using the ‘Quantify’-tool in EaSeq with all normalization settings disabled and exported for analysis in Deseq2 (60). Output, including significance levels and fold change was reimported as a Regionset in EaSeq and visualized in volcano plots. Localization relative to enhancers and LADs were determined using the ‘Coloc’-tool in EaSeq set to measure distances from border to border of the regions, and peaks were further sub-grouped using the ‘Gate’-tool in EaSeq and the derived distances. For each peak, the nearest gene was identified using the ‘Annotate’-tool in EaSeq set to calculate distances from peak centers to TSSes. Coloured volcano plots were generated using the ‘Zscatter’-plot tool in EaSeq with custom adjustment of scales, bin numbers and colour scales based on colouring from http://www.ColorBrewer.org (61). Where nothing else is mentioned plotting and data handling was done using Microsoft Excel and statistical testing and adjustments for multiple testing was performed using R (https://www.R-project.org).

### Mitophagy and autophagy evaluation by immunofluorescence microscopy

To evaluate mitophagy and autophagy, cells were grown for 24 h on coverslips, and 5 h before the end of the experiment the cells were treated alternatively with a control solution (PBS, CTR) or chloroquine 40 μM (CQ, Sigma-aldrich, C6628) to block lysosomes acidification and degradation of autophagy/mitophagy cargos. Staining, visualization, and analysis was achieved as previously reported (62). Briefly, cells were then fixed and permealized by methanol for 10 min at -20°C. After permeabilization, cells were blocked for 1 h with a blocking solution (PBS/normal goat serum 5% v/v, FBA 1% v/v), and then incubated overnight with anti-LC3 (NanoTools, 0231-100/LC3-5F10) and anti-TOM20 (Santa Cruz Biotechnology, sc-11415). Cells were then washed with cold PBS and incubated for 1 h with fluorophore-conjugated secondary antibodies (respectively, Alexa Fluor 647 and 568). Nuclei were stained with 10 μM Hoechst 33342 (Thermo Fisher Scientific). Confocal microscopy experiments were performed by using LSM700 microscope (ZEISS) equipped with ZEN imaging software, and fluorescence images were adjusted for brightness, contrast, and color balance by Fiji ImageJ analysis software (63). We calculated the percentage of mitochondria (TOM20 stained) within LC3-positive puncta using the open-source plugin ComDet v. 0.3.7. Mitophagy rate was calculated by subtracting the % of TOM20 colocalizing with LC3 in control (PBS-treated) cells from the % of TOM20 colocalizing with LC3 in CQ-treated cells (accumulation of mitochondrial particles within autophagosomes in the 5h of the treatment). The co-localization was considered positive if the maximum distance between the center of two particles was ≤2px. Image projection were achieved by summing the fluorescence signal of the central z-stacks (three planes, 0.3 μm) and adjusted for brightness and contrast by Fiji ImageJ software. Autophagy rate was calculated by subtracting the number of LC3 puncta of in control (PBS-treated) cells from number of LC3 puncta of CQ-treated cells.

### Mitochondrial flux analysis

Real time oxygen consumption rates (OCR) were measured using the XFe96 extracellular flux analyser (Seahorse Bioscience). Cells were seeded at 20 000 cells per well in XFe96 cell culture microplates (Seahorse Bioscience) in DMEM medium and incubated overnight. The OCRs were measured in Seahorse assay medium (10 mM glucose, 10 mM pyruvate, at pH 7.4) at 37°C. Different mitochondrial respiratory states were investigated through sequential addition of diverse compounds that modulate or inhibit mitochondrial respiration differently. Firstly, ‘basal OCR’ was measured to indicate cellular respiration under normal physiological conditions. Thereafter, oligomycin (1 μM) [inhibitor of the mitochondrial *F*_0_/*F*_1_ ATP synthase (complex V)] was added to obtain information about mitochondrial ‘proton leak’ (i.e. OCR occurring mainly due to proton leak across the mitochondrial inner membrane) and ‘ATP-linked OCR’ (fraction of the OCR that is directly interconnected with mitochondrial ATP production). Following, the uncoupling agent carbonylcyanide-p-trifluoromethoxyphenylhydrazone (FCCP; 1 μM) was added to acquire the maximum respiratory rate (i.e. ‘maximum capacity’ of the electron transport system). The difference between maximum capacity and basal OCR indicates the ‘reserve capacity’ of the electron transport chain (i.e. the potential capacity of a cell to increase its OCR under energy demanding conditions). Finally, a combination of rotenone (2.5 μM) and antimycin-A (Anti A; 2.5 μM) was added to inhibit the activity of complexes III, and I respectively, in order to obtain non-mitochondrial OCR (‘NM OCR’) (to enable correction for non-mitochondrial sources of OCR). Specifically, the OCR parameters are calculated as follows: Basal OCR = OCR before drugs added - NM OCR; Protein leak = OCR after oligomycin - NM OCR; Maximum Capacity = OCR after FCCP – NM OCR; ATP linked OCR = OCR before drugs added – OCR after oligomycin; reserve capacity = OCR after FCCP - OCR before drugs added. Following the OCR measurements, the cells were stained with Hoechst-33342 (1 μg/ml) and PI (1 μg/ml) for 15 min at 37°C in PBS; cellular viability and cell death were examined using Celigo Cell Imaging Cytometer (Nexcelom Bioscience). Only cells that were stained with Hoechst-33342 and without PI were defined as viable cells; this number was used to correct the OCR values. For a graphical description of the bioenergetic parameters described above, refer to the supplemental section of our previous publication (64).

### Cell viability assay

For viability assays, 20 000 HGPS and normal fibroblasts were plated in 96-well plates in DMEM containing 10% FBS. After 24 h incubation, the cells were washed in PBS and exposed to the indicated stressors (in DMEM without FBS) over a range of doses as indicated. Oxidative DNA damage was induced and sustained by treatment for 6 h with hydrogen peroxide (H_2_O_2_) or 2 h with menadione. At the end of the treatments, the cells were washed with PBS and with 100 μl of fresh medium and then 10 μl of WST-1 (Roche) was added. After 3 h of incubation in WST-1, the absorbance (450 nm – 630 nm) was read to estimate the number of viable cells. WST-1 is a cell proliferation and viability reagent. The stable tetrazolium salt WST-1 is cleaved to a soluble formazan by a complex cellular mechanism that occurs primarily at the cell surface. The amount of formazan dye formed directly correlates to the number of metabolically active cells in the culture.

### Statistical analysis

Statistical comparisons and graphing were performed using GraphPad Prism 6.03 software (La Jolla, CA, USA). Statistical significance was determined by two tailed Student's *t*-test, or, in the case of proliferation assays, two-way ANOVA Sidak's multiple comparisons test. ***P < 0.001, **P < 0.01, *P < 0.05 were considered as statistically significant.

## RESULTS

### Microarray analysis reveals alterations of metabolic processes in *Lmna*^−/−^ MEFs and HGPS fibroblasts

To identify potential links between dysfunction in nuclear lamins and metabolic processes, we mined our previous microarray data from *Lmna*^−/−^ MEFs (NCBI’s Gene Expression Omnibus, accession number GSE120389) (4). We identified several GO terms with *Z* score below –2 that could indicate mitochondrial dysfunction (Supplementary Table S1; highlighted in grey). In addition, we carried out microarray analysis on Normal-1/HGPS-1, Normal-2/HGPS-2 and Normal-3/HGPS-3 fibroblasts. We report the top 10 upregulated and downregulated GO terms (based on the average of the three Normal/HGPS pairs) (Supplementary Table S2). There were no GO terms that specifically indicate mitochondrial dysfunction, however, several GO terms related to transcription and DNA binding were identified as downregulated in HGPS, thus directing us to examine transcriptional regulators or metabolites that link metabolic inputs to gene regulation.

### Lamin A/C depletion leads to impaired mitochondrial bioenergetics, reduced NAD^+^ biosynthesis, lower SIRT1 deacetylase activity and impaired PGC1α-chromatin interaction

**Figure 1.**
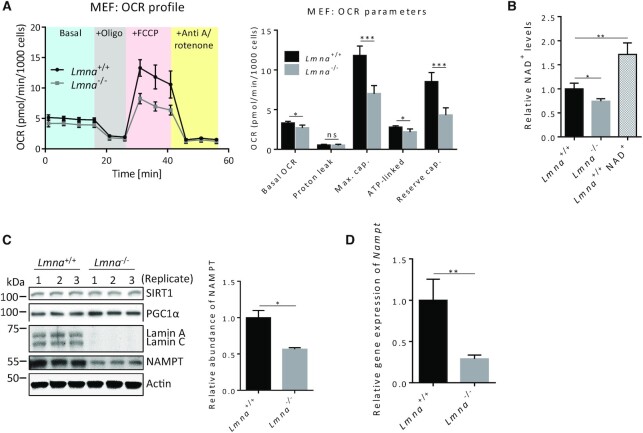
Lamin A/C knockout MEFs exhibit impaired mitochondrial respiration and reduced NAD^+^ biosynthesis. All measurements were performed on *Lmna*^−/−^ MEFs and control (*Lmna*^+/+^) MEFs. (**A**) Seahorse mitochondrial flux analysis. The measurements were used to generate the OCR profile (left) and to calculate OCR parameters (right). (**B**) NAD^+^ levels were measured using an NAD^+^/NADH Assay Kit (Colorimetric). As a positive control, NAD^+^ was added into the medium of the *Lmna*^+/+^ MEFs at 2 mM for 24 h (labeled as *Lmna*^+/+^ NAD^+^). (**C**) Western blotting using antibodies against SIRT1, PGC1α and the NAD^+^ salvage pathway enzyme NAMPT. The NAMPT levels were significantly reduced and band quantification of its abundance is shown. (**D**) *Nampt* mRNA levels, determined by quantitative RT-PCR. Data are presented as mean ± SD (*n* = 3). All P values were calculated using two-sided, unpaired Student's *t*-test; ***P ≤ 0.001, **P ≤ 0.01, *P ≤ 0.05; ns, non-significant; cap., capacity.

Evidence suggests that lamin A/C plays a role in mitochondrial maintenance (21,22). An informative method to assess proper mitochondrial activity is through mitochondrial bioenergetics profiles generated from the Seahorse flux analyzer. The levels of basal OCR (oxygen consumption rate), ATP-linked OCR, maximum capacity and reserve capacity were significantly reduced in the *Lmna*^−/−^ MEFs (Figure 1A and Supplementary Figure S1). Seahorse flux analysis on human U2OS cells upon siRNA-mediated knockdown of lamin A/C (siLamin A/C U2OS) gave results consistent with the MEFs (Supplementary Figure S2). Since the NAD^+^-dependent SIRT1-PGC1α pathway is important in mitochondrial function (33,34) and we observed altered mitochondrial respiration in the absence of lamins A/C, we next measured the NAD^+^ levels in *Lmna*^−/−^ MEFs relative to control MEFs and found that the NAD^+^ levels were reduced (Figure 1B). As a positive control we supplemented the *Lmna*^+/+^ MEFs with 2 mM NAD^+^ for 24 h; this led to a significant increase in cellular NAD^+^ levels. Interestingly, the levels of SIRT1 and of PGC1α were not altered (Figure 1C); however, the levels of the enzyme NAMPT (Figure 1C) and *Nampt* mRNA (Figure 1D) were reduced, in accordance with impaired NAD^+^ biosynthesis. NAMPT abundance was also reduced in MEFs that have siRNA knockdown of lamin A/C (Supplementary Figure S3); thus, a complete removal of lamin A/C is not necessary to elicit reduction in NAMPT abundance. The reduction of the expression and abundance of NAMPT in the absence of lamins appears to be a conserved feature of mammalian cells as lamin A/C-depleted human fibroblasts (siLamin A/C-BJ cells relative to siScr-BJ cells) also exhibited a significant reduction of this protein (Supplementary Figure S4).

**Figure 2.**
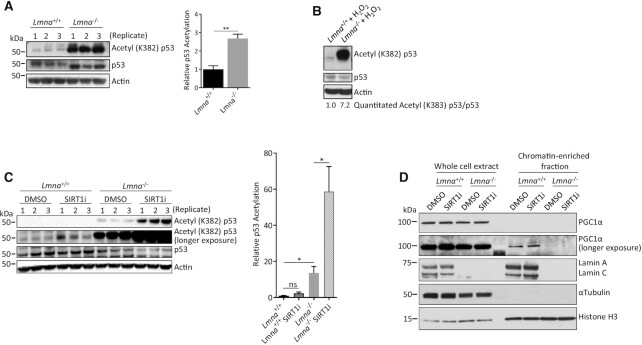
Lamin A/C knockout results in reduced SIRT1 deacetylase activity (higher p53-lysine382 acetylation) and impaired PGC1α-chromatin interaction. (**A**) Western blotting for p53 acetylation in lysates prepared from *Lmna*^−/−^ MEFs and control (*Lmna*^+/+^) MEFs. (**B**) Western blotting to assess the effect of oxidative stress (H_2_O_2_ at 800 μM for 5 h) on p53 acetylation. The relative abundance of acetyl (K382) p53/p53 corrected for actin is written below the image. (**C**) MEFs were treated with the SIRT1 inhibitor selisistat (2 μM for 24 h) and then lysates prepared and immunoblotted for p53 acetylation. The relative p53 acetylation was determined as a ratio of acetylated p53 to normal p53. (**D**) Immunoblotting of whole cell extracts and chromatin-enriched fractions of *Lmna*^−/−^ and *Lmna*^+/+^ MEFs to assess levels of PGC1α in these fractions. The SIRT1 inhibitor was included to enable assessment of SIRT1 dependence. Data are presented as mean ± SD (*n* = 3). Replicates refer to lysates prepared from three separate experiments. All P values were calculated using two-sided, unpaired Student's *t*-test; **P ≤ 0.01, *P ≤ 0.05; ns, non-significant.

SIRT1 deacetylase activity is known to promote mitochondrial biogenesis and respiration (32,34,65). We sought to examine the role of SIRT1 in lamin A/C-regulated mitochondrial function by measuring its activity in lamin A/C-null cells. In *Lmna*^−/−^ MEFs, we found that SIRT1-dependent deacetylation of p53 (Ac-p53K382) was impaired (Figure 2A). Since oxidative stress can cause mitochondrial dysfunction (66), we examined its effect on SIRT1 activity in a lamin A/C-null environment. After treatment with oxidative stress (800 μM H_2_O_2_, 5 h), the SIRT1 deacetylase activity in *Lmna*^−/−^ MEFs was further reduced (relative to *Lmna*^+/+^ MEFs) and the cells accumulated acetyl-p53 (Figure 2B, compared to A), suggesting regulation by oxidative stress. The effect of lamin A/C removal on p53 acetylation was confirmed to be SIRT1-dependent by using a SIRT1 inhibitor (selisistat) (Figure 2C).

The SIRT1 target PGC1α is an important co-activator of key transcription factors in mitochondrial biogenesis. Thus, interaction with gene promoters and co-activating proteins within the chromatin landscape is an important determinant of PGC1α activity. Moreover, PGC1α subcellular distribution is regulated, and its transcriptional activity is promoted, through SIRT1-dependent nuclear accumulation (67). To investigate the regulation of PGC1α by lamin A/C, we assessed the effect of lamin A/C removal on PGC1α-chromatin interaction by examining PGC1α levels in chromatin-enriched (nuclear) fractions. Compared to normal MEFs, there was a much lower, barely detectable, level of PGC1α in chromatin fractions of *Lmna*^−/−^ MEFs (Figure 2D), signifying impaired PGC1α-mediated transcription activity. However, chromatin-associated levels were not affected by SIRT1 inhibition, suggesting that lamin A/C-dependent PGC1α-chromatin interaction is SIRT1-independent.

### Mitochondrial defects and enhanced mitophagy in *Lmna*^−/−^ MEFs

**Figure 3.**
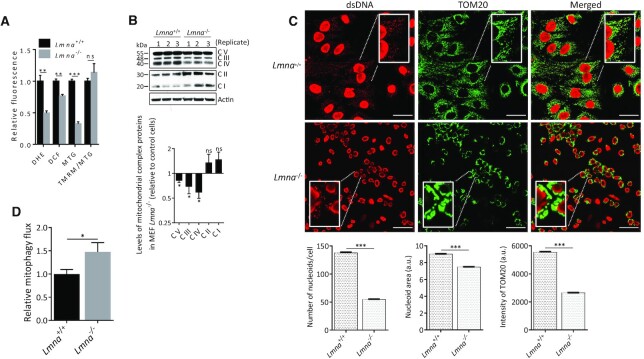
The effect of lamin A/C knockout on ROS levels, mitochondrial mass, OXPHOS components, mtDNA stability and mitophagy. (**A**) Flow cytometry analysis was performed on MEFs (*Lmna*^+/+^, *Lmna*^−/−^). Mitochondrial ROS was estimated from the DHE signal (superoxide indicator). Cellular ROS was estimated from the DCF signal (H_2_DCFDA, indicator of total cellular ROS). Total mitochondria mass was determined using MitoTracker green (MTG), and membrane potential was determined using TMRM. Membrane potential was calculated as TMRM/MTG. Data are presented as mean ± SD (*n* = 3). (**B**) Western blotting was performed using total OXPHOS antibody cocktail to determine the levels of subunits of each of the five OXPHOS complexes (CI-CV). Identity of subunits of each complex: CI = NDUFB8 (NADH dehydrogenase [ubiquinone] 1 beta subcomplex subunit 8), CII = SDHB (Succinate dehydrogenase [ubiquinone] iron-sulfur subunit), CIII = UQCRC2 (Cytochrome b-c1 complex subunit 2), CIV = MTCO1 (mitochondrially encoded cytochrome c oxidase I), CV = vATP5A (ATP synthase, H + transporting, mitochondrial F1 complex, alpha 1). Data are presented as mean ± SD (n = 3). Replicates refer to lysates prepared from three separate experiments. (**C**) High content imaging was performed to determine mitochondrial mass, nucleoid area, and nucleoid counts in *Lmna*^+/+^ MEFs (*n* = 6263 cells) and *Lmna*^−/−^ MEFs (*n* = 10418 cells); mean ± SEM. Cells were fixed and stained with an antibody for mitochondrial outer membrane marker TOM20 for mitochondrial mass estimate, and with anti-dsDNA antibody for detection of nucleoid area and nucleoid counts. (**D**) Mitophagy rate was calculated by subtracting the % of TOM20 co-localizing with LC3 in control (PBS-treated) cells from % of TOM20 co-localizing with LC3 in chloroquine (CQ)-treated cells. Results are shown as the averages ± SEM of 3 independent experiments. All P values were calculated using two-sided, unpaired Student's *t*-test; ***P ≤ 0.001, **P ≤ 0.01, *P ≤ 0.05; ns, non-significant.

To assess lamin A/C-mediated perturbations in mitochondria, we measured the effect of the lamin A/C deletion on ROS, mitochondrial mass and mitochondrial membrane potential (Δψ_m_), using flow cytometry (Figure 3A). The mitochondrial mass-dependent signal of MitoTracker green (MTG) was reduced in *Lmna*^−/−^ MEFs, which is consistent with the less active NAD^+^-SIRT1 signaling. The membrane potential (normalized for mitochondrial mass; TMRM/MTG) was unchanged. Mitochondrial ROS (estimated by quantifying the superoxide-dependent signal of DHE) and whole cell ROS (estimated by the DCF signal) were reduced, consistent with lower mitochondrial mass.

Defects during mitochondrial biogenesis will likely be reflected in perturbations of OXPHOS complexes (68). Thus, the levels of specific subunits in the five mitochondrial membrane complexes were measured, as representatives of each associated complex. This was done by western blotting using a ‘Total OXPHOS’ antibody. We found reduced abundance of CIII, CIV, and CV in *Lmna*^−/−^ MEFs (Figure 3B). We performed the same experiment on siLamin A/C U2OS cells and found that only subunit IV was reduced in this cell model (Supplementary Figure S5), likely due to differences in the internal cellular environment (human versus mouse cells) or the extent of lamin A/C depletion. Notably, this method detects only some specific subunits and so it is possible that other subunits of the five complexes are also altered by lamin A/C depletion.

To support the above flow cytometry data on mitochondrial mass, we performed confocal microscopy and high content imaging to stain for mitochondrial components. We stained the *Lmna*^−/−^ and control MEFs using fluorescent antibodies for the mitochondrial membrane (TOM20; green signal) as an indicator of mitochondrial mass, and simultaneously for double-stranded DNA (dsDNA; red signal) to enable quantification of nucleoid area and numbers (Figure 3C). Interestingly, in the magnified image regions of the *Lmna*^−/−^ MEFs (green signal) one can clearly see much enhanced perinuclear mitochondrial localization, an indication of mislocalized mitochondria. Quantification of all signal intensities showed that TOM20, nucleoid area and nucleoid numbers were all reduced in the *Lmna*^−/−^ MEFs. PCR analysis indicated a reduced abundance of mtDNA (relative to nuclear DNA) (Supplementary Figure S6), thus validating the nucleoid data.

Mitochondrial biogenesis is balanced with mitophagy, together defining mitochondrial turnover (69,70). To our knowledge there is no data linking lamin A/C depletion with altered mitophagy. Using confocal fluorescence microscopy with anti-TOM20 to visualize mitochondria, anti-LC3 to identify autophagosomes, and lysosome inhibitor chloroquine (CQ) (Supplementary Figure S7A), we determined that mitophagy flux was enhanced in *Lmna*^−/−^ MEFs (Figure 3D), whereas autophagy flux was unchanged (Supplementary Figure S7B and C). Since mitophagy is commonly triggered by disrupted membrane potential, which we found to be unchanged by lamin A/C knockout, we speculated that there might be other defects that trigger mitophagy. A recent study showed that casein kinase 2 (CK2) phosphorylates the receptor FUNC1 to inhibit mitophagy in mammalian cells (71). We found that the level of CK2 was reduced in *Lmna*^−/−^ MEFs and siLamin A/C U2OS cells, relative to control cells (Supplementary Figure S7D and E), thus implicating impaired CK2 in promoting receptor-mediated mitophagy in *Lmna*^−/−^ MEFs.

### Effects of PARP1 inhibition and oxidative stress on mitochondrial bioenergetic parameters in *Lmna*^−/−^ MEFs

**Figure 4.**
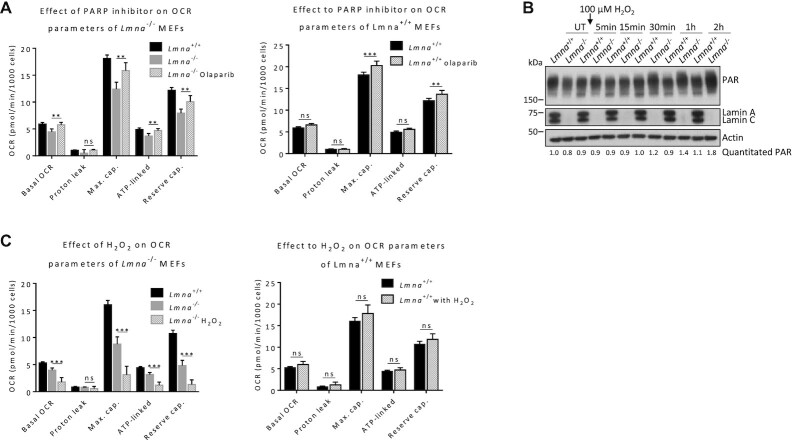
Effect of PARP1 inhibition and oxidative stress on mitochondrial bioenergetic parameters in *Lmna*^−/−^ and *Lmna*^−/−^ MEFs. (**A**) Cells were treated with 2 μM of PARP1 inhibitor olaparib for 2 h and analyzed by the Seahorse flux analyzer. The calculated bioenergetic parameters are shown. (**B**) Cells were treated with 100 μM H_2_O_2_ for 1 h, medium replaced with H_2_O_2_-free medium, and lysates collected at the indicated time points for western blotting; the blots were probed with the antibody against poly(ADP-ribose) (PAR). The relative abundance of PAR corrected for actin is written below the image. (**C**) Cells were treated with 100 μM H_2_O_2_ for 2 h and analyzed by the Seahorse flux analyzer. The calculated bioenergetic parameters are shown. Data are presented as mean ± SD (*n* = 6). All P values were calculated using two-sided, unpaired Student's *t*-test; ***P ≤ 0.001, **P ≤ 0.01; ns, non-significant; cap., capacity; UT, Untreated.

NAD^+^ has emerged as a key regulator of metabolism, stress resistance and longevity. To gain insight into the role of lamin A/C in NAD^+^ signaling under genotoxic stress, we examined the effect of adding an inhibitor (olaparib) of the NAD^+^-consumer (and DNA-damage response protein) PARP1. We chose a treatment condition of 2 μM olaparib for two hours since we found that this inhibits PARylation (PAR formation) in MEFs (Supplementary Figure S8). We observed a significant reversal in all four bioenergetics parameters that were reduced by lamin A/C knockout (basal OCR, maximum cap., ATP-linked OCR and reserve cap.); however, two of those parameters (maximum cap. and reserve cap.) were also enhanced in normal MEFs (Figure 4A). Thus, only basal OCR and ATP-linked OCR were increased selectively in *Lmna*^−/−^ MEFs by olaparib, suggesting an important role for NAD^+^ consumption by PARylation in the impairment of these two parameters in a lamin A/C-null cellular environment. We also tested for a potential rescue of impaired bioenergetics in lamin A/C null MEFs by NAD^+^ supplementation (2 mM NAD^+^ for 24 hours). This treatment condition was chosen based on our existing data showing that it is effective at increasing NAD^+^ in the MEFs (Figure 1B). This treatment resulted in a significant increase in the levels of the maximum capacity and reserve capacity in both normal and *Lmna*^−/−^ MEFs (Supplementary Figure S9); thus, the positive effects of NAD^+^ supplementation on bioenergetics was not restricted to a lamin A/C-null environment.

It is known that NAD^+^-consuming PARylation is triggered by DNA damage (72), and we recently demonstrated that *Lmna*^−/−^ MEFs accumulate oxidative lesions due to less efficient base excision repair (4). Thus, we subjected the normal and lamin A/C-null MEFs to oxidative stress (100 μM H_2_O_2_ treatment) and found that this leads to increasingly pronounced PAR formation (PARylation) in *Lmna*^−/−^ MEFs, and to a lesser degree in normal MEFs, as time progresses (Figure 4B). The same effect of H_2_O_2_ stress on PARylation was observed in siLamin A/C U2OS cells (Supplementary Figure S10). We then assessed the effect of the same concentration of PAR-inducing H_2_O_2_ stress (treatment for period of 2 h) on lamin A/C-dependent bioenergetics. The H_2_O_2_ treatment led to a significant additional reduction in the four parameters already reduced in the *Lmna*^−/−^ MEFs; this oxidative stress had no effect on the levels of the bioenergetic parameters in normal *Lmna*^+/+^ MEFs (Figure 4C). This data suggests that lamin A/C depletion leads to accumulation of DNA damage-induced PARylation (and thus presumably increased NAD^+^ consumption), which in turn worsens bioenergetics.

### Chromatin accessibility is altered in *Lmna*^−/−^ MEFs

Nuclear lamins have important roles in epigenetics, chromatin organization, DNA replication and transcription (6). A recent study revealed that skin fibroblasts from HGPS patients exhibit chromatin accessibility changes that are enriched in lamina-associated domains (LADs) and specifically that the chromatin associated with LADs have a propensity to undergo relaxation in HGPS cells (73). They also found that the epigenetic deregulation of LADs is associated with HGPS-specific gene expression changes. Thus, we decided to pursue a complementary analysis with the *Lmna*^−/−^ and *Lmna*^+/+^ MEFs. Further rationale for such analysis comes from our data in this current study showing that SIRT1 deacetylase activity is impaired by *Lmna* deletion in MEFs, combined with the fact that SIRT1 can modulate chromatin compaction and transcription through direct deacetylation of histones, as well as by promoting alterations in the methylation of histones and DNA (74).

**Figure 5.**
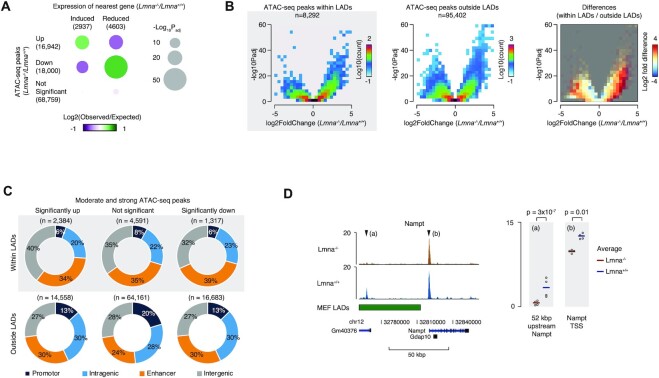
Chromatin accessibility is altered by lamin A/C depletion. (**A**) Bubble plots, showing the relative frequencies of ATAC-seq peaks with combinations of ATAC-seq changes (Y-axis) and transcriptional changes (X-axis), in *Lmna*^−/−^ compared to *Lmna*^+/+^ MEFs, relative to the frequencies expected by change if ATAC-seq and transcriptional changes are unrelated. Bubble sizes illustrate -log10 P values Benjamini-Hochberg adjusted for multiple testing, and the color reflects the number of observed peaks in each category log_2_ normalized to the expected number. (**B**) Volcano plots, showing differences (X-axis) and significance (Y-axis) between ATAC-seq signal in *Lmna*^−/−^ compared to *Lmna*^+/+^ MEFs. The color reflects the density of peaks with a given combination of change and *P* value. (**C**) Donut plots, showing the genome-wide localization of different subtypes of peaks relative to gene features and enhancers. A peak was assigned as ‘promotor’ if it was located within 1 kb of a TSS, ‘intragenic’ if it overlapped with any gene body, or ‘enhancer’, if it was located less than 1 kb from an enhancer midpoint in prioritized order. Peaks that were not assigned to any of these categories were assigned as ‘intergenic’. (**D**) Genome-browser tracks of ATAC-seq signal in *Lmna*^−/−^ and *Lmna*^+/+^ MEFs at the *Nampt* locus. Values are FPKM normalized and the four replicates from each condition are rendered transparent and superimposed. ATAC-seq experiments were done on four biological replicates, and *P* values are Benjamini–Hochberg corrected for multiple testing.

We started by mining the microarray data for *Lmna*^−/−^ compared to *Lmna*^+/+^ MEFs that was performed in our previous study (4) and testing the reported gene expression levels for correlation with chromatin accessibility. To do this, we performed ATAC-seq, identified peaks with differential accessibility, annotated ATAC-seq peaks to the nearest transcription start site (TSS), and counted the number of TSSes from significantly altered genes revealed by the microarray analysis. This analysis revealed a strongly significant association between up-regulated ATAC-seq peaks (enhanced accessibility) and induced expression of the nearest gene, as well between down-regulated ATAC-seq peaks (decreased accessibility) and reduced gene expression (Figure 5A).

Next, we examined changes in chromatin accessibility due to *Lmna* deletion by measuring ATAC-seq peaks in *Lmna*^−/−^ MEFs relative to *Lmna*^+/+^ MEFs (Figure 5B). Volcano plots of these differences show that the *Lmna*^−/−^ MEFs display a striking number of ATAC-seq signal changes (peak signals that are either up or down), both within and outside LADs. Moreover, there was a clear overrepresentation of peaks with increased ATAC-seq signal (within and outside the LADs) in the *Lmna*^−/−^ MEFs (see the left and middle plots) and within LADs compared to outside LADs (as summarized in the right plot).

Analysis of genome-wide localization of ATAC-seq peaks (Figure 5C) indicated that chromatin accessibility at gene regulatory elements (promoters and enhancers) was strikingly altered (higher or lower) both within and outside of LADs. In fact, lamin A/C knockout resulted in significantly up or down ATAC-seq signals, many occurring within LADs with 34% of the ‘significantly up’ and 39% of the ‘significantly down’ signals occurring within enhancers. Outside of LADs, the percent up (30%) or down (30%) were slightly lower. In the case of promoters, the percent up or down were slightly higher outside LADs.

Since mRNA expression from the *Nampt* gene was reduced in *Lmna*^−/−^ MEFs, we made genome-browser tracks of ATAC-seq signal at the *Nampt* locus to see if promoter accessibility was altered. This revealed that the transcription start site (TSS) and a putative upstream enhancer, in the non-coding RNA GM40376 upstream of the *Nampt* gene, had a reduced ATAC-seq signal in *Lmna*^−/−^ MEFs (Figure 5D). This suggests that there is higher chromatin compaction within the promoter and enhancer regions of the *Nampt* gene, which may contribute to the reduced gene expression observed. Interestingly, this potential enhancer region of the *Nampt* gene lies within a LAD (Figure 5D, green demarcation). Taken together, our ATAC-seq data provide evidence that the chromatin accessibility landscape is substantially altered in *Lmna*^−/−^ MEFs and that these changes are enriched in LADs (with a significant predominance for chromatin relaxation), consistent with chromatin landscape alteration reported in HGPS cells (73). The *Nampt* gene expression appears to be hindered by chromatin compaction; this is important because the gene product NAMPT is the rate-limiting step in the salvage pathway for NAD^+^ biosynthesis.

### HGPS fibroblasts display impaired bioenergetics, reduced NAD^+^ levels and defects in mitochondrial biogenesis

**Figure 6.**
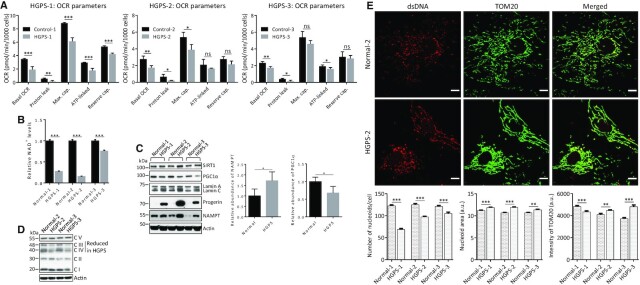
HGPS fibroblasts exhibit impaired mitochondrial respiration, attenuation of NAD^+^ biosynthesis, reduced levels of specific OXPHOS components and lower PGC1α abundance. All measurements were performed on dermal fibroblasts from the indicated HGPS patients and normal counterparts (parent). (**A**) Seahorse mitochondrial flux analysis was performed; the calculated bioenergetic parameters are shown. Data are presented as mean ± SD (*n* = 3). (**B**) NAD^+^ levels were measured using an NAD^+^/NADH Assay Kit (Colorimetric). Data are presented as mean ± SD (*n* = 3). (**C**) Western blotting using antibodies against SIRT1, PGC1α and the NAD^+^ salvage pathway enzyme NAMPT. The PGC1α levels were significantly reduced and NAMPT levels enhanced, as shown graphically as the mean all three Normal/HGPS pairs (mean ± SD). (**D**) Western blotting was performed using total OXPHOS antibody cocktail to determine the levels of subunits of each of the five OXPHOS complexes (CI-CV). Identity of subunits of each complex: CI = NDUFB8 (NADH dehydrogenase [ubiquinone] 1 beta subcomplex subunit 8), CII = SDHB (Succinate dehydrogenase [ubiquinone] iron-sulfur subunit), CIII = UQCRC2 (Cytochrome b-c1 complex subunit 2), CIV = MTCO1 (mitochondrially encoded cytochrome c oxidase I), CV = vATP5A (ATP synthase, H + transporting, mitochondrial F1 complex, alpha 1). (**E**) High content imaging was performed to determine mitochondrial mass, nucleoid area, and nucleoid counts in HGPS fibroblasts (n = 359 cells for HGPS-1, 1183 cells for HGPS-2 and 302 cells for HGPS-3) and in normal fibroblasts (*n* = 613 cells for Normal-1, 250 cells for Normal-2 and 446 cells for Normal-3). Cells were fixed and stained with antibody for mitochondrial outer membrane marker TOM20 to enable an estimate of mitochondrial mass, and with anti-dsDNA antibody for detection of nucleoid area and nucleoid counts. Representative images are shown for Normal-2/HGPS-2 fibroblasts. Data are presented as mean ± SEM. All P values were calculated using two-sided, Student's *t*-test (unpaired except for part C which used paired analysis); ***P ≤ 0.001, **P ≤ 0.01, *P ≤ 0.05; ns, non-significant; cap., capacity.

We hypothesized that the *Lmna* mutation that leads to HGPS, and the ensuing accumulation of the toxic lamin A mutant protein (progerin), will have similar effects on mitochondrial respiration signaling as the lamin A/C depletion. Thus, we performed mitochondrial flux analysis on fibroblasts from HGPS patients and the normal counterparts. As we saw in *Lmna*^−/−^ MEFs, the HGPS cells exhibited attenuated bioenergetic parameters; however, there was some variability among the three sets of Normal/HGPS fibroblasts (Figure 6A). HGPS-1 had reduced levels of all five parameters; HGPS-2 had reductions in basal OCR, proton leak and maximum capacity; HGPS-3 had reduced levels of basal OCR, proton leak and ATP-linked OCR. Thus, the data indicates consistent attenuation in basal OCR and proton leak in dermal fibroblasts from all three different HGPS individuals. NAD^+^ supplementation resulted in significant reversal of the impaired maximum capacity, ATP-linked OCR and reserve capacity in HGPS-1 fibroblasts, and not in the Normal-1 fibroblasts; however, in HGPS-2 none of the impaired parameters were corrected by NAD^+^, and in HGPS-3 the basal OCR and ATP-linked OCR were corrected by NAD^+^ (and not in the Normal-3) (Supplementary Figure S11). Thus, no parameter was corrected in the fibroblasts from all three patients by NAD^+^ supplementation.

Importantly, as with the *Lmna*^−/−^ MEFs, the NAD^+^ levels in the fibroblasts from all three HGPS individuals were significantly reduced (Figure 6B). Thus, it was unexpected to find that NAMPT abundance was enhanced in HGPS cells (Figure 6C). Also, in contrast to the *Lmna*^−/−^ MEF data, the PGC1α protein abundance was altered (reduced) in the HGPS cells (consistently in all 3 patients). As with the *Lmna*^−/−^ MEFs, the SIRT1 level was unchanged in HGPS cells. It is likely that lower PGC1α abundance results in attenuated PGC1α transcription factor co-activation activity on downstream gene targets associated with mitochondrial biogenesis; this could contribute to the observed attenuation in OXPHOS components CIII and CIV (Figure 6D) and nucleoid numbers (Figure 6E) seen in fibroblast from the HGPS individuals. Notably, the mitochondrial network appears hyperfused in the HGPS cells, a manifestation of mitochondrial dysfunction. Interestingly, the nucleoids area was enhanced in the HGPS fibroblasts (Figure 6E). The observed alterations in nucleoids suggest mtDNA stress and potential mtDNA instability that could contribute to mitochondrial dysfunction (75).

### HGPS fibroblasts display reduced efficiency of DNA base excision repair

**Figure 7.**
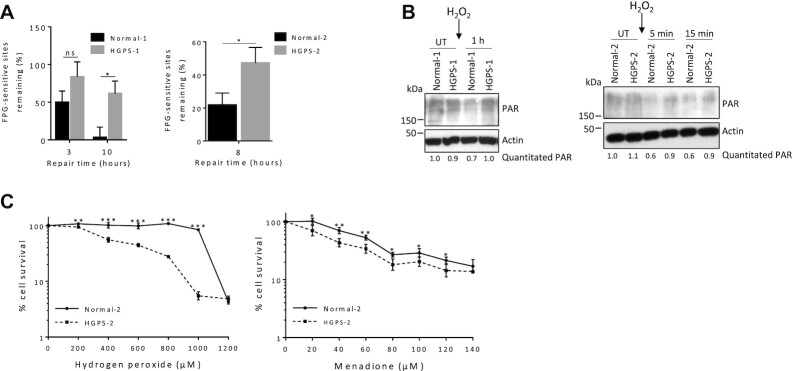
HGPS fibroblasts are defective in DNA base excision repair. (**A**) Comet assays were performed on two pairs of HGPS and normal counterpart fibroblasts, as indicated. The cells were treated with 100 μM H_2_O_2_ for 1 h, the medium replaced with H_2_O_2_-free medium, and cells collected at the indicated time points for comet assay. The FPG enzyme, which cleaves at oxidative lesions, was incorporated into the assay to enable detection of FPG-sensitive sites (estimation of oxidative DNA damage). DNA repair efficiency was expressed as percent of FPG-sensitive sites remaining, at the indicated repair times, relative to 5 min of repair, after correction for the FPG-sensitive sites in untreated cells (*n* = 100 comet tails, mean ± SEM). P values were calculated using two-sided, unpaired Student's *t*-test. (**B**) Normal-1/HGPS-1 and Normal-2/HGPS-2 fibroblasts were treated with 100 μM H_2_O_2_ for 1 h, then the medium replaced with H_2_O_2_-free medium, and then lysates collected at the indicated time points and western blotting performed. The blot was probed with the antibody against poly(ADP-ribose) (PAR). The relative abundance of PAR corrected for actin is written below the image. (**C**) Survival assays were performed on HGPS-2 and its counterpart fibroblasts Normal-2. Oxidative DNA damage was induced and sustained by treatment for 6 h with hydrogen peroxide or 2 h with menadione; WST-1 cell proliferation and viability reagent was used to enable visualization of relative cell viability by absorbance readings (450 nm minus 630 nm). For survival assays, data are presented as mean ± SD (n = 6) and statistical significance was determined by two-way ANOVA Sidak's multiple comparisons test. In all cases, ***P ≤ 0.001, **P ≤ 0.01, *P ≤ 0.05; ns, non-significant; UT, Untreated.

We previously reported that *Lmna*^−/−^ MEFs have impaired DNA base excision repair (BER) of oxidative lesions (4). DNA damage and DNA repair defects are associated with aging, cancer and other diseases (76–78). DNA damage accumulation, as would occur to a greater extent in cells with deficient DNA repair, causes PARP1 activation (PARylation), which consumes NAD^+^. Also, PARP1 activation is known to promote BER (79). Since progerin impairs many activities carried out by lamin A/C, we postulated that BER would also be impaired in HGPS cells. FPG-comet assays were performed on fibroblasts from Normal-1/HGPS-1 and Normal-2/HGPS-2; the extent of FPG-sensitive sites is an estimate for the abundance of oxidative lesions. HGPS cells were significantly less efficient at BER of oxidative lesions generated by H_2_O_2_, for both pairs (Figure 7A). We then tested PAR formation as a marker of DNA damage accumulation. As we saw with the *Lmna*^−/−^ MEFs, the PAR formation was higher in the HGPS cells (relative to their normal counterpart cells) after addition of H_2_O_2,_ (Figure 7B). Interestingly, the PAR response in the normal cells was strikingly reduced after addition of H_2_O_2_. It is plausible that this is due to a robust enhancement of BER enzyme levels in normal human dermal fibroblasts immediately after the addition of H_2_O_2_. In fact, we do observe that core BER enzymes LIG3 and OGG1 quickly become elevated in abundance within five min after the addition of H_2_O_2_ in the normal cells (and not in the HGPS cells) (Supplementary Figure S12); the muted elevation of core BER enzymes in HGPS in response to oxidative stress provides preliminary mechanistic insight for the impaired BER. We then performed cell survival assays since cells that are defective in BER commonly display increased sensitivity to oxidative stress (4). Indeed, HGPS fibroblasts were more sensitive (reduced cell viability) to oxidative stress from H_2_O_2_ and menadione compared to normal cells (Figure 7C). In sum, we used different approaches to provide strong evidence that HGPS are defective in BER and accumulate oxidative DNA damage. This has important implications in the context of PAR formation and NAD^+^ signaling.

## DISCUSSION

**Figure 8.**
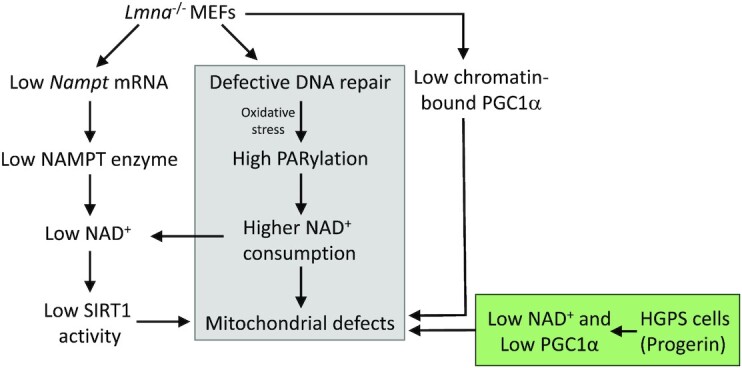
Model depicting the effects of lamin A/C knockout (*Lmna*^−/−^ MEFs) or endogenous progerin expression (HGPS fibroblasts) on mitochondrial function. The grey fill indicates occurrence in both *Lmna*^−/−^ MEFs and in HGPS fibroblasts. The green fill indicates impaired NAD^+^ and PGC1α found in HGPS; specifically, reduced NAD^+^ levels as with *Lmna*^−/−^ MEFs but without accompanying reduction in NAMPT levels, and unlike *Lmna*^−/−^ MEFs the PGC1α protein level was reduced. Both high mitophagy and low SIRT1 activity likely contribute to the observed lower mitochondrial mass we observe in *Lmna*^−/−^ MEFs. Impaired chromatin accessibility at the *Nampt* promoter may contribute to the low *Nampt* mRNA observed in *Lmna*^−/−^ MEFs.

In this study, we made use of two lamin A/C-aberrant cell models to examine the role of lamin A/C in mitochondrial respiration. These two models are quite different in the way that lamin A/C is impaired and they each give insight into the associated diseases. In the case the *Lmna*^−/−^ MEFs, there is no lamin A/C present. The data from this model is particularly important in cancer since many cancer cells have either enhanced or reduced levels of lamins (8). The HGPS cells have a mutation in the *LMNA* gene that leads to expression of the mutant dominant-negative form of the lamin A protein called progerin; there is no change in the levels of normal lamin A or C. The accumulation of progerin leads to deformations of the nuclear membrane, dysregulation of gene expression (18,80), and ultimately to the premature aging characteristics seen in the HGPS children. Thus, the data from the HGPS model is relevant to premature and normal aging. Our study shows that these two types of lamin A/C impairments both lead to reduced NAD^+^ levels and mitochondrial respiratory dysfunction, but apparently by mechanisms that do not wholly overlap, as illustrated in our model (Figure 8). Data suggests that the deleterious effects of progerin are due to the retention of the farnesyl group causing it to become permanently anchored in the nuclear membrane, disrupting proper nuclear scaffolding (18); this implies that progerin is not only directly damaging nuclear and chromatin structures, but also dominantly interfering with the important activities of normal lamin A/C. Mitochondrial dysfunctions, including changes in mtDNA stability, bioenergetics and mitochondrial turnover, all of which we report here, are implicated in cancer and aging (81–83). Insight from out data could help in the development of strategies for treatment of cancer (84,85), aging-associated pathologies (86,87), and progeroid syndromes including HGPS (88). For example, there is evidence that alleviation of HGPS phenotypes can be mediated by promoting recovery of mitochondrial function via combating mitochondrial ROS damage (89–91). Future research directions toward therapeutics should include the use of lamin A-related mouse models, which are categorized as null mutants, point mutants and progeroid mutants (92).

Dysregulation of mitochondrial biogenesis signaling is known to impair the assembly of OXPHOS subunits and mitochondrial bioenergetics (93). Moreover, lower levels of the reduced form of NAD^+^ (i.e. NADH) lead to lower respiratory flux since NADH serves as the primary electron donor in the ETC (94). Four fundamental bioenergetic parameters, namely basal OCR, maximum capacity, reserve capacity and ATP-linked OCR were reduced in *Lmna*^−/−^ MEFs. Basal OCR and proton leak were reduced in HGPS cells. This is consistent with our data that indicated defects in aspects of mitochondrial biogenesis, and with the known regulation of mitochondrial quality control and bioenergetics crosstalk described in Hill *et al.* (93). In brief, the attenuated basal OCR we saw for *Lmna*^−/−^ MEFs and HGPS cells may have been due to the observed reduction in substrate supply (reduced NAD^+^ levels) or the impairments in OXPHOS components; low substrate availability can also impair maximum capacity, reserve capacity and ATP-linked OCR. In the case of *Lmna*^−/−^ MEFs, we saw reduced mitochondrial mass; this may lead to the lower basal OCR, as well as the lower maximum and reserve capacity. The reduced proton leak in HGPS cells is consistent with the low basal OCR. The fact that neither of the two lamin A/C-impaired models exhibited enhanced proton leak implies that the mitochondria are not severely damaged by the reduced lamin A/C levels or the toxic progerin lamin A variant.

Our flow cytometry MitoTracker green measurements revealed that mitochondrial mass is reduced in *Lmna*^−/−^ MEFs. This data is supported by our high-content imaging results. Specifically, the reduction in TOM20 mitochondrial membrane marker and in the nucleoid area and counts indicate a reduction in mitochondrial mass. However, reduced nucleoid area and numbers may also reflect improperly assembled nucleoids or reduced mtDNA replication (95). Importantly, reduction in the number of nucleoids signifies reduced mtDNA polyploidy. Reduced area and numbers of nucleoids can lead to mtDNA instability, since proper packaging and high polyploidy of mtDNA provides protection against deleterious mutations (75,96,97). In the case of HGPS cells, the nucleoid numbers were reduced, and nucleoid area enhanced, suggesting mDNA stress, which can lead to mtDNA instability (75). Studies suggest that mtDNA mutations are associated with premature aging; specifically, it has been reported that mice created to have a mitochondrial DNA polymerase proof-reading deficiency display both elevated mtDNA mutation rate and premature aging phenotypes (98,99). The reduced mitochondrial mass in *Lmna*^−/−^ MEFs plausibly contributes to the reduced ROS levels.

Mitophagy was enhanced in *Lmna*^−/−^ MEFs, which may contribute to the reduced mitochondrial mass. Accumulating evidence suggests that mitophagy is important in limiting ROS damage to OXPHOS components and the associated mtDNA mutations. The processes underlying the regulation of mitophagy depend on several factors, including the integrity of mtDNA, OXPHOS chain activity, and the interaction and regulation of the autophagic machinery (93). It is typically triggered by disrupted mitochondria membrane potential; however, this was not altered in the *Lmna*^−/−^ MEFs. Our data suggests that the mitophagy was possibly triggered by the low CK2 levels in these cells. This kinase also promotes DNA BER (100,101), and thus its reduced abundance might also contribute to the impaired BER efficiency.

Importantly, both lamin A/C-impaired cellular systems showed reduced NAD^+^ levels. Interestingly, a study reported that metabolism of NAD^+^ is altered in *Lmna* cardiomyopathy: the *Nampt* mRNA expression, NAMPT protein expression and NAD^+^ levels were all reduced in the hearts of mice with cardiomyopathy caused by a *Lmna* mutation (102). Our data links lamin A/C levels and progerin to the known cellular regulatory activities of NAD^+^. An alteration in the NAD^+^ pool size can influence diverse cellular functions, including metabolic pathways, DNA repair, cell cycle control, chromatin remodeling, cellular senescence and immune cell function (103,104), and contribute to various neurodegenerative disorders, aging, and tumorigenesis (103,105,106).

The mechanism for reduced NAD^+^ level in *Lmna*^−/−^ MEFs appears to be reduced transcription of the *Nampt* gene; our ATAC-seq data suggests that reduced access to transcription factors at the *Nampt* promoter may contribute to the lower *Nampt* mRNA and thus the lower levels of the NAMPT enzyme. NAD^+^ levels are determined by the balance between NAD^+^ biosynthesis and consumption (26). The salvage pathway for NAD^+^ biosynthesis uses NAMPT as a key enzyme. Surprisingly, NAMPT abundance was enhanced in HGPS cells, possibly as a compensatory adaption to low NAD^+^. A recent study showed that, following renal ischaemia, PGC1α^−/−^ mice develop local deficiency of the NAD^+^ precursor nicotinamide (NAM) (39). Since NAMPT converts NAM to nicotinamide mononucleotide (NMN) in the NAD + salvage pathway, reduced NAM in the HGPS cells could lead to a compensatory increase in NAMPT. It is possible that the reduced NAD^+^ we observe in the *Lmna*^−/−^ MEFs and HGPS cells may reflect high NAD^+^ consumption, possibly accompanied by attenuation of one of the other two NAD^+^ biosynthesis pathways. In fact, data suggests that impaired PGC1α (which we observed in both lamin A/C-aberrant cell models) may attenuate the enzymes that synthesize NAD^+^ de novo from amino acids under oxidative stress (38,39).

The mitochondrial membrane OXPHOS complexes connect bioenergetics to mitochondrial biogenesis and turnover processes (107). Defects in assembly of the complexes can arise from mutations in individual subunits, mutations in assembly factors for the complexes, mtDNA depletion, or as a result of physiological or pathological changes. The observed reduction in levels of the designated subunits suggests that there are defects in complex III, IV and V in *Lmna*^−/−^ MEFs, complex IV in siLamin A/C U2OS cells and complex III and IV in HGPS cells. Thus, complex IV (subunit COX1), which is a proton pump driven my electron transport, was the only complex impaired in all three of these lamin A/C-impaired cellular systems. Our HGPS results are consistent with data from a recent study (24) in which HGPS skin fibroblasts were shown to display reduced levels of COX1. In that study, it was also reported that cytochrome c and the complex V protein β-ATPase were reduced in the HGPS fibroblasts. In sum, these data indicate that lamin A/C-depletion and progerin expression lead to impaired proton pumping across the inner mitochondrial membrane, in agreement with our mitochondrial flux bioenergetics data.

Our ATAC-seq data demonstrate that MEFs with deletion of the *Lmna* gene exhibit considerable changes in chromatin accessibility (both higher and lower chromatin compaction), with a tendency for reduced compaction. Moreover, changes in accessibility were significantly associated with the aberrant gene expression patterns in *Lmna*^−/−^ MEFs. Importantly, the alterations in the chromatin accessibility landscape within LADs were clearly inclined towards gain of accessibility compared to regions outside LADs. This is consistent with data reported for skin fibroblasts from HGPS patients (73). We speculate that the mechanism for the above chromatin compaction changes in a lamin A/C-depleted environment involves the reduction in SIRT1 deacetylase activity (since this will diminish histone deacetylation), combined with altered lamina-chromatin interactions.

There were variable results in measurements obtained using fibroblasts from the three different HGPS patients, a phenomenon that somewhat limited the general conclusions regarding mitochondrial flux and mitochondrial mass. The observed differences in progerin expression levels might have an impact on these measurements. Our western blotting data showed that HGPS-2 had high progerin expression compared to HGPS-1 and HGPS-3. The patients also vary in age (from age three to 13), and there will be accompanying changes in hormones of these children and in the epigenetic and signaling milieu of their cells.

The insights gained in this study are summarized as a schematic model (Figure 8). In sum, both lamin A/C deletion and progerin expression lead to a decrease in NAD^+^ levels. In *Lmna*^−/−^ MEFs, a contributing mechanism appears to be reduced NAMPT enzyme expression. Both lamin A/C-defective models exhibited reduced capability of PGC1α: in *Lmna*^−/−^ MEFs the important regulatory activity of PGC1α interaction with chromatin is impaired, and in HGPS cells the abundance of PGC1α in reduced. Further studies are needed to assess how removal of lamin A/C, or progerin expression, leads to these effects on PGC1α; however, it is plausible that the altered chromatin accessibility landscape plays a role. In addition, the *Lmna*^−/−^ MEFs exhibited a reduction in SIRT1 deacetylase activity, in line with reduced levels of SIRT1 substrate NAD^+^. Interestingly, lamin A has been shown to interact with SIRT1 and activate its deacetylase activity (108), which is consistent with our findings. We also showed that BER of DNA is less efficient in HGPS cells, consistent with our previous study showing that *Lmna*^−/−^ MEFs are defective in BER (4). Impaired BER and the ensuing DNA damage accumulation may contribute to the higher levels of PARP1 activation (PARylation) relative to control cells, and thereby enhance relative PARylation-dependent NAD^+^ consumption. In this model, all disrupted pathways lead to mitochondrial dysfunction that encompass nucleoid perturbations, impairments in specific OXPHOS components, and ultimately to attenuated bioenergetics. The novel data from this study add to accumulating evidence that lamins are important in cancer and aging. Thus, our study could help inspire future strategies that would promote healthier aging, combat cancer, and mitigate age-associated processes that are displayed by HGPS patients.

## DATA AVAILABILITY

The microarray and ATAC-seq data sets used in this study have been deposited in NCBI’s Gene Expression Omnibus (GEO) (109) under accession numbers GSE124465 and GSE186677, respectively.

The R code for differential ATAC-seq enrichment analysis is shown in Supplementary Materials and Methods.

## Supplementary Material

gkac741_Supplemental_File

## Notes

Present address: Scott Maynard, Pathology Department, University of Michigan, Ann Arbor, MI 48109, USA.

Present address: Muhammad Shoaib, Department of Biology, SBA School of Science and Engineering, Lahore University of Management Sciences, Lahore 54792 Pakistan.

## References

[1] Burke B. , Stewart C.L. The nuclear lamins: flexibility in function. Nat. Rev. Mol. Cell Biol. 2013; 14:13–24. 23212477 10.1038/nrm3488

[2] Shimi T. , Pfleghaar K., Kojima S., Pack C.G., Solovei I., Goldman A.E., Adam S.A., Shumaker D.K., Kinjo M., Cremer T. et al. The A- and B-type nuclear lamin networks: microdomains involved in chromatin organization and transcription. Genes Dev. 2008; 22:3409–3421. 19141474 10.1101/gad.1735208 PMC2607069

[3] Gonzalo S. DNA damage and lamins. Adv. Exp. Med. Biol. 2014; 773:377–399. 24563357 10.1007/978-1-4899-8032-8_17 PMC4081481

[4] Maynard S. , Keijzers G., Akbari M., Ezra M.B., Hall A., Morevati M., Scheibye-Knudsen M., Gonzalo S., Bartek J., Bohr V.A. Lamin A/C promotes DNA base excision repair. Nucleic Acids Res. 2019; 47:11709–11728. 31647095 10.1093/nar/gkz912 PMC7145687

[5] Dubik N. , Mai S. Lamin A/C: function in normal and tumor cells. Cancers (Basel.). 2020; 12:3688. 10.3390/cancers12123688 PMC7764147 33316938

[6] Dechat T. , Pfleghaar K., Sengupta K., Shimi T., Shumaker D.K., Solimando L., Goldman R.D. Nuclear lamins: major factors in the structural organization and function of the nucleus and chromatin. Genes Dev. 2008; 22:832–853. 18381888 10.1101/gad.1652708 PMC2732390

[7] Milon B.C. , Cheng H., Tselebrovsky M.V., Lavrov S.A., Nenasheva V.V., Mikhaleva E.A., Shevelyov Y.Y., Nurminsky D.I. Role of histone deacetylases in gene regulation at nuclear lamina. PLoS One. 2012; 7:e49692. 23226217 10.1371/journal.pone.0049692 PMC3511463

[8] Broers J.L. , Ramaekers F.C. The role of the nuclear lamina in cancer and apoptosis. Adv. Exp. Med. Biol. 2014; 773:27–48. 24563342 10.1007/978-1-4899-8032-8_2

[9] Irianto J. , Pfeifer C.R., Ivanovska I.L., Swift J., Discher D.E. Nuclear lamins in cancer. Cell Mol Bioeng. 2016; 9:258–267. 27570565 10.1007/s12195-016-0437-8 PMC4999255

[10] Kong L. , Schafer G., Bu H., Zhang Y., Zhang Y., Klocker H. Lamin A/C protein is overexpressed in tissue-invading prostate cancer and promotes prostate cancer cell growth, migration and invasion through the PI3K/AKT/PTEN pathway. Carcinogenesis. 2012; 33:751–759. 22301279 10.1093/carcin/bgs022

[11] Bonne G. , Di Barletta M.R., Varnous S., Becane H.M., Hammouda E.H., Merlini L., Muntoni F., Greenberg C.R., Gary F., Urtizberea J.A. et al. Mutations in the gene encoding lamin A/C cause autosomal dominant emery-dreifuss muscular dystrophy. Nat. Genet. 1999; 21:285–288. 10080180 10.1038/6799

[12] De Sandre-Giovannoli A. , Bernard R., Cau P., Navarro C., Amiel J., Boccaccio I., Lyonnet S., Stewart C.L., Munnich A., Le M.M. et al. Lamin a truncation in hutchinson-gilford progeria. Science. 2003; 300:2055. 12702809 10.1126/science.1084125

[13] Gonzalez-Suarez I. , Redwood A.B., Gonzalo S. Loss of A-type lamins and genomic instability. Cell Cycle. 2009; 8:3860–3865. 19901537 10.4161/cc.8.23.10092

[14] Kozlov S. , Mounkes L., Cutler D., Sullivan T., Hernandez L., Levy N., Rottman J., Stewart C.L. Mutations in the mouse lmna gene causing progeria, muscular dystrophy and cardiomyopathy. Novartis. Found. Symp. 2005; 264:246–258. 15773758

[15] Liu B. , Wang J., Chan K.M., Tjia W.M., Deng W., Guan X., Huang J.D., Li K.M., Chau P.Y., Chen D.J. et al. Genomic instability in laminopathy-based premature aging. Nat. Med. 2005; 11:780–785. 15980864 10.1038/nm1266

[16] Mounkes L.C. , Burke B., Stewart C.L. The A-type lamins: nuclear structural proteins as a focus for muscular dystrophy and cardiovascular diseases. Trends Cardiovasc. Med. 2001; 11:280–285. 11709282 10.1016/s1050-1738(01)00126-8

[17] McClintock D. , Ratner D., Lokuge M., Owens D.M., Gordon L.B., Collins F.S., Djabali K. The mutant form of lamin a that causes hutchinson-gilford progeria is a biomarker of cellular aging in human skin. PLoS One. 2007; 2:e1269. 18060063 10.1371/journal.pone.0001269 PMC2092390

[18] Gonzalo S. , Kreienkamp R., Askjaer P. Hutchinson-Gilford progeria syndrome: a premature aging disease caused by LMNA gene mutations. Ageing Res. Rev. 2016; 33:18–29. 27374873 10.1016/j.arr.2016.06.007 PMC5195863

[19] Walker B.R. , Moraes C.T. Nuclear-Mitochondrial interactions. Biomolecules. 2022; 12:427. 35327619 10.3390/biom12030427 PMC8946195

[20] Quiros P.M. , Mottis A., Auwerx J. Mitonuclear communication in homeostasis and stress. Nat. Rev. Mol. Cell Biol. 2016; 17:213–226. 26956194 10.1038/nrm.2016.23

[21] Sieprath T. , Corne T.D., Nooteboom M., Grootaert C., Rajkovic A., Buysschaert B., Robijns J., Broers J.L., Ramaekers F.C., Koopman W.J. et al. Sustained accumulation of prelamin a and depletion of lamin A/C both cause oxidative stress and mitochondrial dysfunction but induce different cell fates. Nucleus. 2015; 6:236–246. 25996284 10.1080/19491034.2015.1050568 PMC4615646

[22] Lopez-Mejia I.C. , de Toledo M., Chavey C., Lapasset L., Cavelier P., Lopez-Herrera C., Chebli K., Fort P., Beranger G., Fajas L. et al. Antagonistic functions of LMNA isoforms in energy expenditure and lifespan. EMBO Rep. 2014; 15:529–539. 24639560 10.1002/embr.201338126 PMC4210101

[23] Mateos J. , Landeira-Abia A., Fafian-Labora J.A., Fernandez-Pernas P., Lesende-Rodriguez I., Fernandez-Puente P., Fernandez-Moreno M., Delmiro A., Martin M.A., Blanco F.J. et al. iTRAQ-based analysis of progerin expression reveals mitochondrial dysfunction, reactive oxygen species accumulation and altered proteostasis. Stem. Cell Res. Ther. 2015; 6:119. 26066325 10.1186/s13287-015-0110-5 PMC4487579

[24] Rivera-Torres J. , Acin-Perez R., Cabezas-Sanchez P., Osorio F.G., Gonzalez-Gomez C., Megias D., Camara C., Lopez-Otin C., Enriquez J.A., Luque-Garcia J.L. et al. Identification of mitochondrial dysfunction in hutchinson-gilford progeria syndrome through use of stable isotope labeling with amino acids in cell culture. J. Proteomics. 2013; 91:466–477. 23969228 10.1016/j.jprot.2013.08.008

[25] Feric M. , Demarest T.G., Tian J., Croteau D.L., Bohr V.A., Misteli T. Self-assembly of multi-component mitochondrial nucleoids via phase separation. EMBO J. 2021; 40:e107165. 33619770 10.15252/embj.2020107165 PMC7957436

[26] Fang E.F. , Lautrup S., Hou Y., Demarest T.G., Croteau D.L., Mattson M.P., Bohr V.A. NAD(+) in aging: molecular mechanisms and translational implications. Trends Mol. Med. 2017; 23:899–916. 28899755 10.1016/j.molmed.2017.08.001 PMC7494058

[27] Yoshino J. , Mills K.F., Yoon M.J., Imai S. Nicotinamide mononucleotide, a key NAD(+) intermediate, treats the pathophysiology of diet- and age-induced diabetes in mice. Cell Metab. 2011; 14:528–536. 21982712 10.1016/j.cmet.2011.08.014 PMC3204926

[28] Hegedus C. , Virag L. Inputs and outputs of poly(ADP-ribosyl)ation: relevance to oxidative stress. Redox Biol. 2014; 2:978–982. 25460733 10.1016/j.redox.2014.08.003 PMC4215470

[29] Wei H. , Yu X. Functions of PARylation in DNA damage repair pathways. Genomics Proteomics Bioinformatics. 2016; 14:131–139. 27240471 10.1016/j.gpb.2016.05.001 PMC4936651

[30] Masutani M. , Fujimori H. Poly(ADP-ribosyl)ation in carcinogenesis. Mol. Aspects. Med. 2013; 34:1202–1216. 23714734 10.1016/j.mam.2013.05.003

[31] Zhang N. , Sauve A.A. Regulatory effects of NAD(+) metabolic pathways on sirtuin activity. Prog. Mol. Biol. Transl. Sci. 2018; 154:71–104. 29413178 10.1016/bs.pmbts.2017.11.012

[32] Nogueiras R. , Habegger K.M., Chaudhary N., Finan B., Banks A.S., Dietrich M.O., Horvath T.L., Sinclair D.A., Pfluger P.T., Tschop M.H. Sirtuin 1 and sirtuin 3: physiological modulators of metabolism. Physiol. Rev. 2012; 92:1479–1514. 22811431 10.1152/physrev.00022.2011 PMC3746174

[33] Houtkooper R.H. , Pirinen E., Auwerx J. Sirtuins as regulators of metabolism and healthspan. Nat. Rev. Mol. Cell Biol. 2012; 13:225–238. 22395773 10.1038/nrm3293 PMC4872805

[34] Nemoto S. , Fergusson M.M., Finkel T. SIRT1 functionally interacts with the metabolic regulator and transcriptional coactivator PGC-1{alpha}. J. Biol. Chem. 2005; 280:16456–16460. 15716268 10.1074/jbc.M501485200

[35] Guarente L. Sirtuins in aging and disease. Cold Spring Harb. Symp. Quant. Biol. 2007; 72:483–488. 18419308 10.1101/sqb.2007.72.024

[36] Kaarniranta K. , Kajdanek J., Morawiec J., Pawlowska E., Blasiak J. PGC-1alpha protects RPE cells of the aging retina against oxidative stress-induced degeneration through the regulation of senescence and mitochondrial quality control. The significance for AMD pathogenesis. Int. J. Mol. Sci. 2018; 19:2317. 10.3390/ijms19082317 PMC6121367 30087287

[37] Youdim M.B. , Oh Y.J. Promise of neurorestoration and mitochondrial biogenesis in parkinson's disease with multi target drugs: an alternative to stem cell therapy. Exp. Neurobiol. 2013; 22:167–172. 24167412 10.5607/en.2013.22.3.167 PMC3807004

[38] Koh J.H. , Kim J.Y. Role of PGC-1alpha in the mitochondrial NAD(+) pool in metabolic diseases. Int. J. Mol. Sci. 2021; 22:4558. 33925372 10.3390/ijms22094558 PMC8123861

[39] Tran M.T. , Zsengeller Z.K., Berg A.H., Khankin E.V., Bhasin M.K., Kim W., Clish C.B., Stillman I.E., Karumanchi S.A., Rhee E.P. et al. PGC1alpha drives NAD biosynthesis linking oxidative metabolism to renal protection. Nature. 2016; 531:528–532. 26982719 10.1038/nature17184 PMC4909121

[40] Sullivan T. , Escalante-Alcalde D., Bhatt H., Anver M., Bhat N., Nagashima K., Stewart C.L., Burke B. Loss of A-type lamin expression compromises nuclear envelope integrity leading to muscular dystrophy. J. Cell Biol. 1999; 147:913–920. 10579712 10.1083/jcb.147.5.913 PMC2169344

[41] Toledo L.I. , Altmeyer M., Rask M.B., Lukas C., Larsen D.H., Povlsen L.K., Bekker-Jensen S., Mailand N., Bartek J., Lukas J. ATR prohibits replication catastrophe by preventing global exhaustion of RPA. Cell. 2013; 155:1088–1103. 24267891 10.1016/j.cell.2013.10.043

[42] Karakaya A. , Jaruga P., Bohr V.A., Grollman A.P., Dizdaroglu M. Kinetics of excision of purine lesions from DNA by escherichia coli fpg protein. Nucleic Acids Res. 1997; 25:474–479. 9016584 10.1093/nar/25.3.474 PMC146462

[43] Buenrostro J.D. , Wu B., Chang H.Y., Greenleaf W.J. ATAC-seq: a method for assaying chromatin accessibility genome-wide. Curr. Protoc. Mol. Biol. 2015; 109:21.29.1–21.29.9. 10.1002/0471142727.mb2129s109 PMC4374986 25559105

[44] Andrews S. Babraham Bioinformatics. 2010; UK Babraham Institute, Cambridge.

[45] Wingett S.W. , Andrews S. FastQ screen: a tool for multi-genome mapping and quality control. F1000Res. 2018; 7:1338. 30254741 10.12688/f1000research.15931.1 PMC6124377

[46] Ewels P. , Magnusson M., Lundin S., Kaller M. MultiQC: summarize analysis results for multiple tools and samples in a single report. Bioinformatics. 2016; 32:3047–3048. 27312411 10.1093/bioinformatics/btw354 PMC5039924

[47] Shoaib M. , Walter D., Gillespie P.J., Izard F., Fahrenkrog B., Lleres D., Lerdrup M., Johansen J.V., Hansen K., Julien E. et al. Histone H4K20 methylation mediated chromatin compaction threshold ensures genome integrity by limiting DNA replication licensing. Nat. Commun. 2018; 9:3704. 30209253 10.1038/s41467-018-06066-8 PMC6135857

[48] Bolger A.M. , Lohse M., Usadel B. Trimmomatic: a flexible trimmer for illumina sequence data. Bioinformatics. 2014; 30:2114–2120. 24695404 10.1093/bioinformatics/btu170 PMC4103590

[49] Langmead B. , Salzberg S.L. Fast gapped-read alignment with bowtie 2. Nat. Methods. 2012; 9:357–359. 22388286 10.1038/nmeth.1923 PMC3322381

[50] Li H. , Handsaker B., Wysoker A., Fennell T., Ruan J., Homer N., Marth G., Abecasis G., Durbin R. 1000 Genome Project Data Processing Subgroup The sequence alignment/map format and SAMtools. Bioinformatics. 2009; 25:2078–2079. 19505943 10.1093/bioinformatics/btp352 PMC2723002

[51] Quinlan A.R. , Hall I.M. BEDTools: a flexible suite of utilities for comparing genomic features. Bioinformatics. 2010; 26:841–842. 20110278 10.1093/bioinformatics/btq033 PMC2832824

[52] Buenrostro J.D. , Giresi P.G., Zaba L.C., Chang H.Y., Greenleaf W.J. Transposition of native chromatin for fast and sensitive epigenomic profiling of open chromatin, DNA-binding proteins and nucleosome position. Nat. Methods. 2013; 10:1213–1218. 24097267 10.1038/nmeth.2688 PMC3959825

[53] Lerdrup M. , Johansen J.V., Agrawal-Singh S., Hansen K An interactive environment for agile analysis and visualization of chip-sequencing data. Nat. Struct. Mol. Biol. 2016; 23:349–357. 26926434 10.1038/nsmb.3180

[54] Zhang Y. , Liu T., Meyer C.A., Eeckhoute J., Johnson D.S., Bernstein B.E., Nusbaum C., Myers R.M., Brown M., Li W. et al. Model-based analysis of chip-Seq (MACS). Genome Biol. 2008; 9:R137. 18798982 10.1186/gb-2008-9-9-r137 PMC2592715

[55] O’Leary N.A. , Wright M.W., Brister J.R., Ciufo S., Haddad D., McVeigh R., Rajput B., Robbertse B., Smith-White B., Ako-Adjei D. et al. Reference sequence (RefSeq) database at NCBI: current status, taxonomic expansion, and functional annotation. Nucleic Acids Res. 2016; 44:D733–D745. 26553804 10.1093/nar/gkv1189 PMC4702849

[56] Peric-Hupkes D. , Meuleman W., Pagie L., Bruggeman S.W., Solovei I., Brugman W., Graf S., Flicek P., Kerkhoven R.M., van Lohuizen M. et al. Molecular maps of the reorganization of genome-nuclear lamina interactions during differentiation. Mol. Cell. 2010; 38:603–613. 20513434 10.1016/j.molcel.2010.03.016 PMC5975946

[57] Shen Y. , Yue F., McCleary D.F., Ye Z., Edsall L., Kuan S., Wagner U., Dixon J., Lee L., Lobanenkov V.V. et al. A map of the cis-regulatory sequences in the mouse genome. Nature. 2012; 488:116–120. 22763441 10.1038/nature11243 PMC4041622

[58] Kent W.J. , Sugnet C.W., Furey T.S., Roskin K.M., Pringle T.H., Zahler A.M., Haussler D. The human genome browser at UCSC. Genome Res. 2002; 12:996–1006. 12045153 10.1101/gr.229102 PMC186604

[59] Lerdrup M. , Hansen K. User-Friendly and interactive analysis of chip-Seq data using easeq. Methods Mol. Biol. 2020; 2117:35–63. 31960371 10.1007/978-1-0716-0301-7_2

[60] Love M.I. , Huber W., Anders S. Moderated estimation of fold change and dispersion for RNA-seq data with DESeq2. Genome Biol. 2014; 15:550. 25516281 10.1186/s13059-014-0550-8 PMC4302049

[61] Harrower M. , Brewer C. ColorBrewer.org: an online tool for selecting colour schemes for maps. Cartographic J. The. 2003; 40:27–37.

[62] Cirotti C. , Rizza S., Giglio P., Poerio N., Allega M.F., Claps G., Pecorari C., Lee J.H., Benassi B., Barila D. et al. Redox activation of ATM enhances GSNOR translation to sustain mitophagy and tolerance to oxidative stress. EMBO Rep. 2021; 22:e50500. 33245190 10.15252/embr.202050500 PMC7788447

[63] Schindelin J. , Arganda-Carreras I., Frise E., Kaynig V., Longair M., Pietzsch T., Preibisch S., Rueden C., Saalfeld S., Schmid B. et al. Fiji: an open-source platform for biological-image analysis. Nat. Methods. 2012; 9:676–682. 22743772 10.1038/nmeth.2019 PMC3855844

[64] Maynard S. , Hejl A.M., Dinh T.S., Keijzers G., Hansen A.M., Desler C., Moreno-Villanueva M., Burkle A., Rasmussen L.J., Waldemar G. et al. Defective mitochondrial respiration, altered dNTP pools and reduced AP endonuclease 1 activity in peripheral blood mononuclear cells of alzheimer's disease patients. Aging (Albany. NY). 2015; 7:793–815. 26539816 10.18632/aging.100810 PMC4637207

[65] Tang B.L. Sirt1 and the mitochondria. Mol. Cells. 2016; 39:87–95. 26831453 10.14348/molcells.2016.2318 PMC4757807

[66] Slimen I.B. , Najar T., Ghram A., Dabbebi H., Ben Mrad M., Abdrabbah M. Reactive oxygen species, heat stress and oxidative-induced mitochondrial damage. A review. Int. J. Hyperthermia. 2014; 30:513–523. 25354680 10.3109/02656736.2014.971446

[67] Anderson R.M. , Barger J.L., Edwards M.G., Braun K.H., O’Connor C.E., Prolla T.A., Weindruch R. Dynamic regulation of PGC-1alpha localization and turnover implicates mitochondrial adaptation in calorie restriction and the stress response. Aging Cell. 2008; 7:101–111. 18031569 10.1111/j.1474-9726.2007.00357.x PMC2253697

[68] Ryan M.T. , Hoogenraad N.J. Mitochondrial-nuclear communications. Annu. Rev. Biochem. 2007; 76:701–722. 17227225 10.1146/annurev.biochem.76.052305.091720

[69] Ma K. , Chen G., Li W., Kepp O., Zhu Y., Chen Q. 2020) Mitophagy, mitochondrial homeostasis, and cell fate. Front Cell Dev. Biol. 8:467. 32671064 10.3389/fcell.2020.00467 PMC7326955

[70] Melser S. , Lavie J., Benard G. Mitochondrial degradation and energy metabolism. Biochim. Biophys. Acta. 2015; 1853:2812–2821. 25979837 10.1016/j.bbamcr.2015.05.010

[71] Chen G. , Han Z., Feng D., Chen Y., Chen L., Wu H., Huang L., Zhou C., Cai X., Fu C. et al. A regulatory signaling loop comprising the PGAM5 phosphatase and CK2 controls receptor-mediated mitophagy. Mol. Cell. 2014; 54:362–377. 24746696 10.1016/j.molcel.2014.02.034

[72] Burkle A. Poly(ADP-ribosyl)ation: a posttranslational protein modification linked with genome protection and mammalian longevity. Biogerontology. 2000; 1:41–46. 11707919 10.1023/a:1010089924898

[73] Kohler F. , Bormann F., Raddatz G., Gutekunst J., Corless S., Musch T., Lonsdorf A.S., Erhardt S., Lyko F., Rodriguez-Paredes M. Epigenetic deregulation of lamina-associated domains in hutchinson-gilford progeria syndrome. Genome Med. 2020; 12:46. 32450911 10.1186/s13073-020-00749-y PMC7249329

[74] Zhang T. , Kraus W.L. SIRT1-dependent regulation of chromatin and transcription: linking NAD(+) metabolism and signaling to the control of cellular functions. Biochim. Biophys. Acta. 2010; 1804:1666–1675. 19879981 10.1016/j.bbapap.2009.10.022 PMC2886162

[75] West A.P. , Khoury-Hanold W., Staron M., Tal M.C., Pineda C.M., Lang S.M., Bestwick M., Duguay B.A., Raimundo N., MacDuff D.A. et al. Mitochondrial DNA stress primes the antiviral innate immune response. Nature. 2015; 520:553–557. 25642965 10.1038/nature14156 PMC4409480

[76] Maynard S. , Fang E.F., Scheibye-Knudsen M., Croteau D.L., Bohr V.A. DNA damage, DNA repair, aging, and neurodegeneration. Cold Spring Harb. Perspect. Med. 2015; 5:a025130. 26385091 10.1101/cshperspect.a025130 PMC4588127

[77] Maynard S. , Schurman S.H., Harboe C., de Souza-Pinto N.C., Bohr V.A. Base excision repair of oxidative DNA damage and association with cancer and aging. Carcinogenesis. 2009; 30:2–10. 18978338 10.1093/carcin/bgn250 PMC2639036

[78] Whitaker A.M. , Schaich M.A., Smith M.R., Flynn T.S., Freudenthal B.D. Base excision repair of oxidative DNA damage: from mechanism to disease. Front. Biosci. (Landmark Ed.). 2017; 22:1493–1522. 28199214 10.2741/4555 PMC5567671

[79] Dantzer F. , Schreiber V., Niedergang C., Trucco C., Flatter E., De La Rubia G., Oliver J., Rolli V., Menissier-de Murcia J., de Murcia G. Involvement of poly(ADP-ribose) polymerase in base excision repair. Biochimie. 1999; 81:69–75. 10214912 10.1016/s0300-9084(99)80040-6

[80] Vidak S. , Foisner R. Molecular insights into the premature aging disease progeria. Histochem. Cell Biol. 2016; 145:401–417. 26847180 10.1007/s00418-016-1411-1 PMC4796323

[81] Moro L. Mitochondrial dysfunction in aging and cancer. J. Clin. Med. 2019; 8:1983–1998. 10.3390/jcm8111983 PMC6912717 31731601

[82] Porporato P.E. , Filigheddu N., Pedro J.M.B., Kroemer G., Galluzzi L. Mitochondrial metabolism and cancer. Cell Res. 2018; 28:265–280. 29219147 10.1038/cr.2017.155 PMC5835768

[83] Palikaras K. , Daskalaki I., Markaki M., Tavernarakis N. Mitophagy and age-related pathologies: development of new therapeutics by targeting mitochondrial turnover. Pharmacol. Ther. 2017; 178:157–174. 28461251 10.1016/j.pharmthera.2017.04.005

[84] Johri A. Disentangling mitochondria in alzheimer's disease. Int. J. Mol. Sci. 2021; 22:11520–11548. 34768950 10.3390/ijms222111520 PMC8583788

[85] Kalyanaraman B. , Cheng G., Hardy M., Ouari O., Lopez M., Joseph J., Zielonka J., Dwinell M.B. A review of the basics of mitochondrial bioenergetics, metabolism, and related signaling pathways in cancer cells: therapeutic targeting of tumor mitochondria with lipophilic cationic compounds. Redox. Biol. 2018; 14:316–327. 29017115 10.1016/j.redox.2017.09.020 PMC5633086

[86] Bielas J. , Herbst A., Widjaja K., Hui J., Aiken J.M., McKenzie D., Miller R.A., Brooks S.V., Wanagat J. Long term rapamycin treatment improves mitochondrial DNA quality in aging mice. Exp. Gerontol. 2018; 106:125–131. 29486228 10.1016/j.exger.2018.02.021 PMC5911406

[87] Ozkurede U. , Miller R.A. Improved mitochondrial stress response in long-lived snell dwarf mice. Aging Cell. 2019; 18:e13030. 31423721 10.1111/acel.13030 PMC6826134

[88] Navarro C.L. , Cau P., Levy N. Molecular bases of progeroid syndromes. Hum. Mol. Genet. 2006; 15:R151–R161. 16987878 10.1093/hmg/ddl214

[89] Kang H.T. , Park J.T., Choi K., Choi H.J.C., Jung C.W., Kim G.R., Lee Y.S., Park S.C. Chemical screening identifies ROCK as a target for recovering mitochondrial function in hutchinson-gilford progeria syndrome. Aging Cell. 2017; 16:541–550. 28317242 10.1111/acel.12584 PMC5418208

[90] Richards S.A. , Muter J., Ritchie P., Lattanzi G., Hutchison C.J. The accumulation of un-repairable DNA damage in laminopathy progeria fibroblasts is caused by ROS generation and is prevented by treatment with N-acetyl cysteine. Hum. Mol. Genet. 2011; 20:3997–4004. 21807766 10.1093/hmg/ddr327

[91] Xiong Z.M. , Choi J.Y., Wang K., Zhang H., Tariq Z., Wu D., Ko E., LaDana C., Sesaki H., Cao K. Methylene blue alleviates nuclear and mitochondrial abnormalities in progeria. Aging Cell. 2016; 15:279–290. 26663466 10.1111/acel.12434 PMC4783354

[92] Zhang H. , Kieckhaefer J.E., Cao K. Mouse models of laminopathies. Aging Cell. 2013; 12:2–10. 23095062 10.1111/acel.12021

[93] Hill B.G. , Benavides G.A., Lancaster J.R. Jr, Ballinger S., Dell’Italia L., Jianhua Z., Darley-Usmar V.M Integration of cellular bioenergetics with mitochondrial quality control and autophagy. Biol. Chem. 2012; 393:1485–1512. 23092819 10.1515/hsz-2012-0198 PMC3594552

[94] Canto C. , Menzies K.J., Auwerx J. NAD(+) metabolism and the control of energy homeostasis: a balancing act between mitochondria and the nucleus. Cell Metab. 2015; 22:31–53. 26118927 10.1016/j.cmet.2015.05.023 PMC4487780

[95] Sasaki T. , Sato Y., Higashiyama T., Sasaki N. Live imaging reveals the dynamics and regulation of mitochondrial nucleoids during the cell cycle in Fucci2-hela cells. Sci. Rep. 2017; 7:11257. 28900194 10.1038/s41598-017-10843-8 PMC5595809

[96] Vasileiou P.V.S. , Mourouzis I., Pantos C. Principal aspects regarding the maintenance of mammalian mitochondrial genome integrity. Int. J. Mol. Sci. 2017; 18:1821–1841. 10.3390/ijms18081821 PMC5578207 28829360

[97] Bogenhagen D.F. Mitochondrial DNA nucleoid structure. Biochim. Biophys. Acta. 2012; 1819:914–920. 22142616 10.1016/j.bbagrm.2011.11.005

[98] Kujoth G.C. , Hiona A., Pugh T.D., Someya S., Panzer K., Wohlgemuth S.E., Hofer T., Seo A.Y., Sullivan R., Jobling W.A. et al. Mitochondrial DNA mutations, oxidative stress, and apoptosis in mammalian aging. Science. 2005; 309:481–484. 16020738 10.1126/science.1112125

[99] Trifunovic A. , Wredenberg A., Falkenberg M., Spelbrink J.N., Rovio A.T., Bruder C.E., Bohlooly Y.M., Gidlof S., Oldfors A., Wibom R. et al. Premature ageing in mice expressing defective mitochondrial DNA polymerase. Nature. 2004; 429:417–423. 15164064 10.1038/nature02517

[100] Strom C.E. , Mortusewicz O., Finch D., Parsons J.L., Lagerqvist A., Johansson F., Schultz N., Erixon K., Dianov G.L., Helleday T. CK2 phosphorylation of XRCC1 facilitates dissociation from DNA and single-strand break formation during base excision repair. DNA Repair (Amst.). 2011; 10:961–969. 21840775 10.1016/j.dnarep.2011.07.004

[101] Parsons J.L. , Dianova II, Finch D., Tait P.S., Strom C.E., Helleday T., Dianov G.L XRCC1 phosphorylation by CK2 is required for its stability and efficient DNA repair. DNA Repair (Amst.). 2010; 9:835–841. 20471329 10.1016/j.dnarep.2010.04.008

[102] Vignier N. , Chatzifrangkeskou M., Morales Rodriguez B., Mericskay M., Mougenot N., Wahbi K., Bonne G., Muchir A. Rescue of biosynthesis of nicotinamide adenine dinucleotide protects the heart in cardiomyopathy caused by lamin A/C gene mutation. Hum. Mol. Genet. 2018; 27:3870–3880. 30053027 10.1093/hmg/ddy278

[103] Covarrubias A.J. , Perrone R., Grozio A., Verdin E. NAD(+) metabolism and its roles in cellular processes during ageing. Nat. Rev. Mol. Cell. Biol. 2021; 22:119–141. 33353981 10.1038/s41580-020-00313-x PMC7963035

[104] Stromland O. , Niere M., Nikiforov A.A., VanLinden M.R., Heiland I., Ziegler M. Keeping the balance in NAD metabolism. Biochem. Soc. Trans. 2019; 47:119–130. 30626706 10.1042/BST20180417

[105] Amjad S. , Nisar S., Bhat A.A., Shah A.R., Frenneaux M.P., Fakhro K., Haris M., Reddy R., Patay Z., Baur J. et al. Role of NAD(+) in regulating cellular and metabolic signaling pathways. Mol. Metab. 2021; 49:101195. 33609766 10.1016/j.molmet.2021.101195 PMC7973386

[106] Hou Y. , Dan X., Babbar M., Wei Y., Hasselbalch S.G., Croteau D.L., Bohr V.A. Ageing as a risk factor for neurodegenerative disease. Nat. Rev. Neurol. 2019; 15:565–581. 31501588 10.1038/s41582-019-0244-7

[107] Pfanner N. , Warscheid B., Wiedemann N. Mitochondrial proteins: from biogenesis to functional networks. Nat. Rev. Mol. Cell. Biol. 2019; 20:267–284. 30626975 10.1038/s41580-018-0092-0 PMC6684368

[108] Liu B. , Ghosh S., Yang X., Zheng H., Liu X., Wang Z., Jin G., Zheng B., Kennedy B.K., Suh Y. et al. Resveratrol rescues SIRT1-dependent adult stem cell decline and alleviates progeroid features in laminopathy-based progeria. Cell Metab. 2012; 16:738–750. 23217256 10.1016/j.cmet.2012.11.007

[109] Edgar R. , Domrachev M., Lash A.E. Gene expression omnibus: NCBI gene expression and hybridization array data repository. Nucleic Acids Res. 2002; 30:207–210. 11752295 10.1093/nar/30.1.207 PMC99122

