# Supplementary material for: Lamin A/C impairments cause mitochondrial dysfunction by attenuating PGC1α and the NAMPT-NAD+ pathway
Source: Nucleic Acids Res. 2022 Sep 13;50(17):9948–65. doi: 10.1093/nar/gkac741 (PMC9508839; doi:10.1093/nar/gkac741)
Supplement: gkac741_Supplemental_File [file gkac741_supplemental_file.pdf]

SUPPLEMENTARY MATERIALS AND METHODS

RNA interference

U2OS cells, BJ cells or *Lmna*$^{+/+}$ MEFs, growing in 60 mm dishes in DMEM medium supplemented with 10% FBS, were transfected with 25 nM siRNA oligo using DharmaFECT 1 transfection reagent (Dharmacon, T-2001-02) according to the manufacturer's protocol. The siRNAs (ON-TARGETplus) were chemically synthesized by Dharmacon. The target sequence for siLamin A/C in humans (Dharmacon, J-004978-05) is 5′-GAAGGAGGGUGACCUGAUA-3′. The target sequence for siLamin A/C in mouse (Dharmacon, J-040758-05-0005) is 5′-GGAAGCAGCGAGAGUUUGA-3′. The negative control (scrambled siRNA, designed to target no known genes in human, mouse or rat) was ON-TARGETplus non-targeting number #1 (Dharmacon, D-001810-010). After 24 h incubation, the transfection medium was replaced with fresh culture medium. The cells were harvested, or used for further experiments, 72 h after transfection.

PCR for mtDNA abundance

Total RNA samples were isolated using RNeasy Mini Kit (Qiagen Sciences) according to the manufacturer's protocol. First strand cDNA was synthesized from 2 µg of total RNA with random hexamer primers using High-Capacity cDNA Reverse Transcription kit (Applied Biosystems). qRT-PCR was performed using the ABI Prism 7300 system (Applied Biosystems) and SYBR Select Master Mix containing SYBR Green dye (Applied Biosystems). The relative quantity of cDNA was estimated by the DDCT method. Data was normalized to nuclear DNA. The primers were purchased from Eurofins Genomics and designed according to West AP et al. (1). The primers sequences used for the mouse mtDNA targeted the *m.mtDNA Dloop 1* gene and are as follows:
Forward: 5´-AATCTACCATCCTCCGTGAAACC-3´
Reverse: 5´-TCAGTTTAGCTACCCCCAAGTTTAA-3´.
The primers used for nuclear DNA targeted the *m.nucDNA Tert* gene and are as follows:
Forward: 5´-CTAGCTCATGTGTCAAGACCCTCTT-3´
Reverse: 5´-GCCAGCACGTTTCTCTC GTT-3´.

Antibodies

The following antibodies were used solely in the Supplementary section, for western blotting: αOGG1 (ab124741; Abcam), LIG3 (sc-135883; Santa Cruz), Casein kinase-2 (CK2) (ab76025; Abcam), PARP1 (614302; Nordic Biosite).

R code for differential ATAC-seq enrichment analysis

```
if (!requireNamespace("BiocManager", quietly = TRUE))
  install.packages("BiocManager")
BiocManager::install("DESeq2")
library("DESeq2")

setwd("[INSERT PATH HERE]")
cold <- read.table("coldata.txt", header = TRUE)
cts <-  read.table("peaks_for_de.txt", header = TRUE, row.names = "ID")

dds <- DESeqDataSetFromMatrix(countData = cts, colData = cold, design= ~ condition)
dds <- DESeq(dds)
resultsNames(dds)
res <- results(dds, name="condition_WT_vs_LAC")
write.table(res, "mod_strong_peaks_deseq2.txt", sep = "\t", dec = ",")
```

Workflow of Quantitative Image-Based Cytometry (QIBC) for nucleoid and TOM 20 analysis.

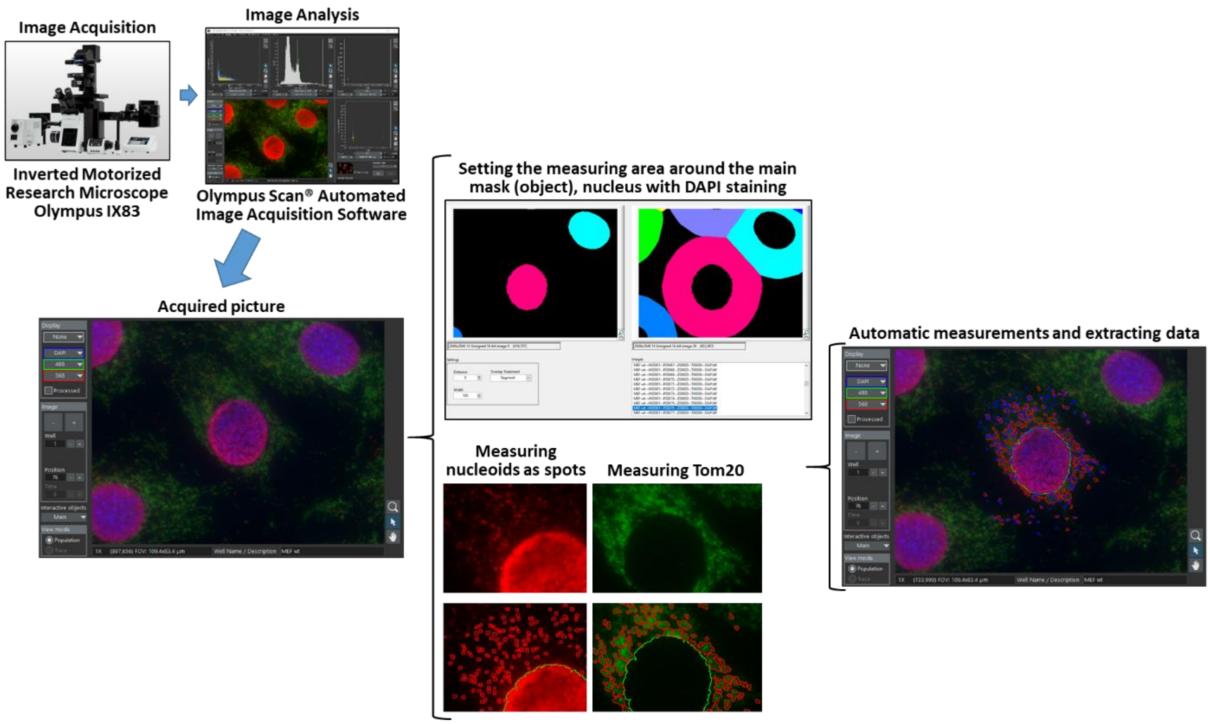

References

1. West, A.P., Khoury-Hanold, W., Staron, M., Tal, M.C., Pineda, C.M., Lang, S.M., Bestwick, M., Duguay, B.A., Raimundo, N., MacDuff, D.A. *et al.* (2015) Mitochondrial DNA stress primes the antiviral innate immune response. *Nature*, **520**, 553-557.

SUPPLEMENTARY TABLES

Supplementary Table S1. Mined gene ontology (GO) terms for *Lmna*$^{-/-}$ versus *Lmna*$^{+/+}$ MEFs

Gene Ontology (GO) Term	Z-score
GO0005634 NUCLEUS	6.405166071
GO0003723 RNA BINDING	6.056850171
GO0006397 MRNA PROCESSING	4.754405865
GO0006350 TRANSCRIPTION	4.571858026
GO0003676 NUCLEIC ACID BINDING	4.502049004
GO0008380 RNA SPLICING	4.39184625
GO0003677 DNA BINDING	4.154068297
GO0009887 ORGAN MORPHOGENESIS	3.939451662
GO0051028 MRNA TRANSPORT	3.921510086
GO0003899 DNA DIRECTED RNA POLYMERASE ACTIVITY	3.9005054
GO0030529 RIBONUCLEOPROTEIN COMPLEX	3.871640069
GO0006364 RRNA PROCESSING	3.610286634
GO0065002 INTRACELLULAR PROTEIN TRANSPORT ACROSS A	3.548790997
GO0006355 REGULATION OF TRANSCRIPTION DNA DEPENDE	3.484741379
GO0005681 SPLICEOSOME	3.427594962
GO0006406 MRNA EXPORT FROM NUCLEUS	3.279575981
GO0009968 NEGATIVE REGULATION OF SIGNAL TRANSDUCTI	3.237757627
GO0004386 HELICASE ACTIVITY	3.196940211
GO0008168 METHYLTRANSFERASE ACTIVITY	2.796281486
GO0000398 NUCLEAR MRNA SPLICING VIA SPLICEOSOME	2.766280473
GO0043234 PROTEIN COMPLEX	2.697147131
GO0007067 MITOSIS	2.532088186
GO0000166 NUCLEOTIDE BINDING	2.431791882
GO0006281 DNA REPAIR	2.362503986
GO0006511 UBIQUITIN DEPENDENT PROTEIN CATABOLIC PR	2.317928886
GO0007059 CHROMOSOME SEGREGATION	2.302482471
GO0008026 ATP DEPENDENT HELICASE ACTIVITY	2.301256211
GO0004221 UBIQUITIN THIOLESTERASE ACTIVITY	2.236350264
GO0051301 CELL DIVISION	2.229621604
GO0005762 MITOCHONDRIAL LARGE RIBOSOMAL SUBUNIT	2.218447828
GO0015934 LARGE RIBOSOMAL SUBUNIT	2.212268832
GO0003735 STRUCTURAL CONSTITUENT OF RIBOSOME	2.153727133
GO0005840 RIBOSOME	2.088809541
GO0000002 MITOCHONDRIAL GENOME MAINTENANCE	2.054294833
GO0030122 AP 2 ADAPTOR COMPLEX	-2.011348606

GO0005044 SCAVENGER RECEPTOR ACTIVITY	-2.036395378
GO0030048 ACTIN FILAMENT BASED MOVEMENT	-2.092668901
GO0005887 INTEGRAL TO PLASMA MEMBRANE	-2.108916817
GO0005080 PROTEIN KINASE C BINDING	-2.132705117
GO0030593 NEUTROPHIL CHEMOTAXIS	-2.402837388
GO0010008 ENDOSOME MEMBRANE	-2.434721281
GO0016459 MYOSIN COMPLEX	-2.443017664
GO0006927 TRANSFORMED CELL APOPTOSIS	-2.462483397
GO0030036 ACTIN CYTOSKELETON ORGANIZATION AND BIOG	-2.492677047
GO0019992 DIACYLGLYCEROL BINDING	-2.526860801
GO0016798 HYDROLASE ACTIVITY ACTING ON GLYCOSYL B	-2.566001791
GO0019370 LEUKOTRIENE BIOSYNTHETIC PROCESS	-2.568123421
GO0006955 IMMUNE RESPONSE	-2.610341018
GO0042981 REGULATION OF APOPTOSIS	-2.644625001
GO0005794 GOLGI APPARATUS	-2.91215026
GO0015629 ACTIN CYTOSKELETON	-3.003891047
GO0050880 REGULATION OF BLOOD VESSEL SIZE	-3.104198285
GO0003774 MOTOR ACTIVITY	-3.191196403
GO0004030 ALDEHYDE DEHYDROGENASE NAD(P)+ ACTIVIT	-3.387727434
GO0016491 OXIDOREDUCTASE ACTIVITY	-3.389634083
GO0003779 ACTIN BINDING	-3.533522519
GO0004720 PROTEIN LYSINE 6 OXIDASE ACTIVITY	-3.66688923
GO0016021 INTEGRAL TO MEMBRANE	-3.74733971
GO0006954 INFLAMMATORY RESPONSE	-3.848746553
GO0006936 MUSCLE CONTRACTION	-4.103796708
GO0008152 METABOLIC PROCESS	-4.215669497
GO0016020 MEMBRANE	-4.244745043
GO0005856 CYTOSKELETON	-4.343032902
GO0005783 ENDOPLASMIC RETICULUM	-4.853657107
GO0005604 BASEMENT MEMBRANE	-5.191387248
GO0005615 EXTRACELLULAR SPACE	-6.367806254
GO0005509 CALCIUM ION BINDING	-7.024102194

Microarray analysis was performed on *Lmna* knockout MEFs (*Lmna*$^{-/-}$ MEFs) compared to wild-type MEFs (*Lmna*$^{+/+}$ MEFs) followed by Gene ontology (GO) analysis of the downregulated and upregulated genes. This data was mined from our previous analysis in Maynard et al. (PMID: 31647095). The GO terms highlighted in grey indicate potential defects in mitochondrial function.

Supplementary Table S2. Gene ontology (GO) terms in HGPS fibroblasts compared to control fibroblasts. TOP 10 upregulated and downregulated GO terms

Gene Ontology (GO) Term	Z-score Pair 1	Z-score Pair 2	Z-score Pair 3	Ave Z-score
GO0008243 PLASMINOGEN ACTIVATOR ACTIVITY	11.7624523	15.74369734	5.552302187	11.01948394
GO0005587 COLLAGEN TYPE IV	9.03917112	11.12335852	5.465491824	8.542673823
GO0005200 STRUCTURAL CONSTITUENT OF CYTOSKELETON	6.04604211	7.653012994	7.795292075	7.164782393
GO0008307 STRUCTURAL CONSTITUENT OF MUSCLE	8.620589406	5.850742728	4.666948086	6.37942674
GO0005198 STRUCTURAL MOLECULE ACTIVITY	4.515108483	6.671243773	6.36955526	5.851969172
GO0030502 NEGATIVE REGULATION OF BONE MINERALIZATI	3.908977876	6.426076598	7.125512647	5.820189041
GO0031674 I BAND	4.977628037	4.347205683	7.978416697	5.767750139
GO0006096 GLYCOLYSIS	4.57682745	8.005250807	4.149983201	5.577353819
GO0005882 INTERMEDIATE FILAMENT	4.071211475	6.712434626	5.261456012	5.348367371
GO0007010 CYTOSKELETON ORGANIZATION AND BIOGENESIS	3.509794215	5.102283597	5.897218108	4.836431973
GO0006350 TRANSCRIPTION*	-5.109341622	-7.121887047	-9.408625904	-7.213284858
GO0005501 RETINOID BINDING	-8.729762943	-9.443627138	-2.394662984	-6.856017688
GO0003676 NUCLEIC ACID BINDING*	-5.71426498	-6.310532921	-7.859428107	-6.628075336
GO0006355 REGULATION OF TRANSCRIPTION DNA DEPENDE*	-4.945052326	-6.699279722	-8.157375975	-6.600569341
GO0003677 DNA BINDING*	-4.905116426	-6.37727419	-7.475423469	-6.252604695
GO0005634 NUCLEUS	-5.511085395	-4.802449826	-8.150443591	-6.154659604
GO0045087 INNATE IMMUNE RESPONSE	-3.391815116	-7.093731753	-7.236549439	-5.907365436
GO0006066 ALCOHOL METABOLIC PROCESS	-4.541105441	-4.122537892	-8.40336666	-5.689003331
GO0004551 NUCLEOTIDE DIPHOSPHATASE ACTIVITY	-4.199416454	-5.248372018	-7.144989238	-5.530925903
GO0008270 ZINC ION BINDING	-5.431776729	-5.188161025	-5.821603555	-5.48051377

Microarray analysis was performed on HGPS and control (normal) fibroblasts, followed by and Gene ontology (GO) analysis of the downregulated and upregulated genes. Pairs 1, 2 and 3 are as follows (denoted as HGPS/Normal): AG03198/AG03258 (pair 1), AG03513/AG03512 (pair 2) and AG06917/AG06299 (pair 3).
*Suggests that HGPS cells have strong negative alteration in transcription at the level of transcription factors binding to DNA

SUPPLEMENTARY FIGURES

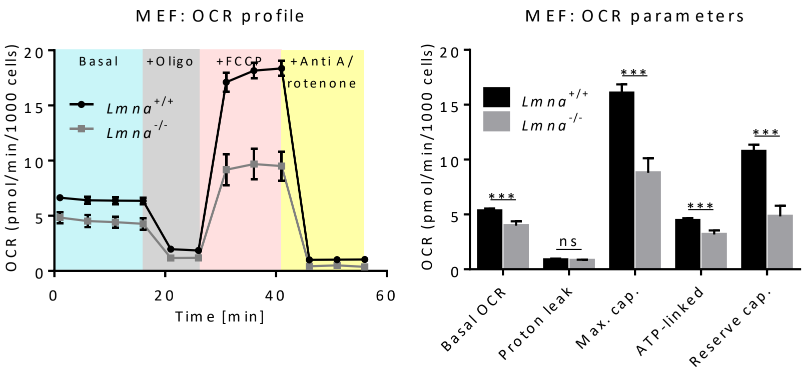

Supplementary Figure S1. Seahorse XF flux analysis verifying that *Lmna* knockout in MEFs reduces multiple bioenergetics parameters. The oxygen consumption rate (OCR) profile (left) and the calculated OCR parameters (right) are shown. Data are presented as sample mean ± SD (n = 6). All P values were calculated using two-sided, unpaired Student's *t*-test; ***P ≤ 0.001; ns, non-significant. cap., capacity.

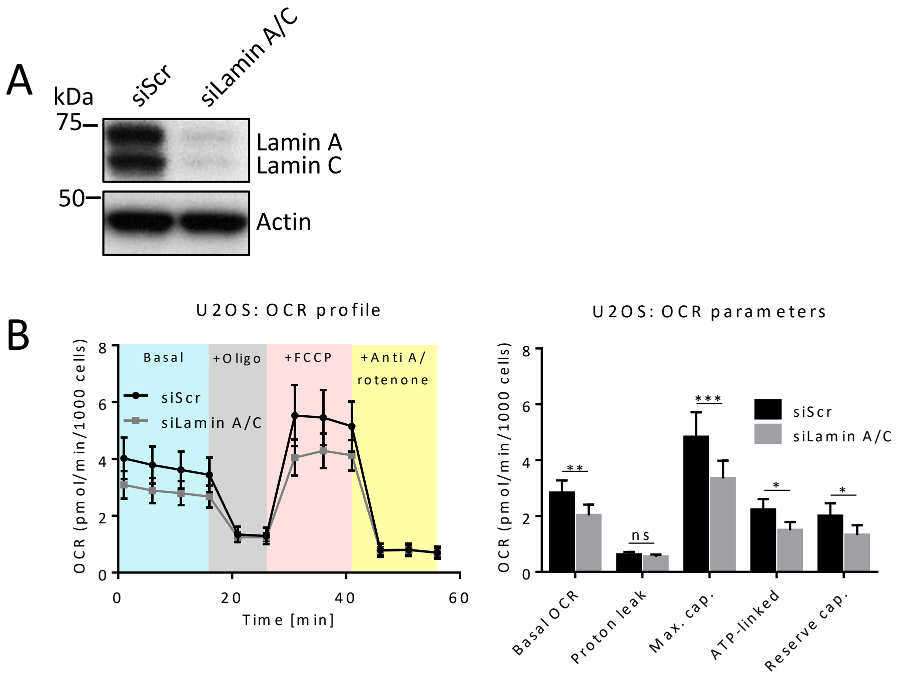

Supplementary Figure S2. Seahorse XF flux analysis showing that lamin A/C knockdown in U2OS cells leads to a reduction in multiple bioenergetics parameters. (A) siRNA knockdown of lamin A/C in U2OS cells. The target sequence for siLamin A/C in humans (Dharmacon ON TARGETplus, J-004978-05) is 5′-GAAGGAGGGUGACCUGAUA-3′. (B) The oxygen consumption rate (OCR) profile (left) and the calculated OCR parameters (right) are shown. Data are presented as sample mean ± SD (n = 6). All P values were calculated using two-sided, unpaired Student’s *t*-test; ***P ≤ 0.001, **P ≤ 0.01, *P ≤ 0.05; ns, non-significant. cap., capacity.

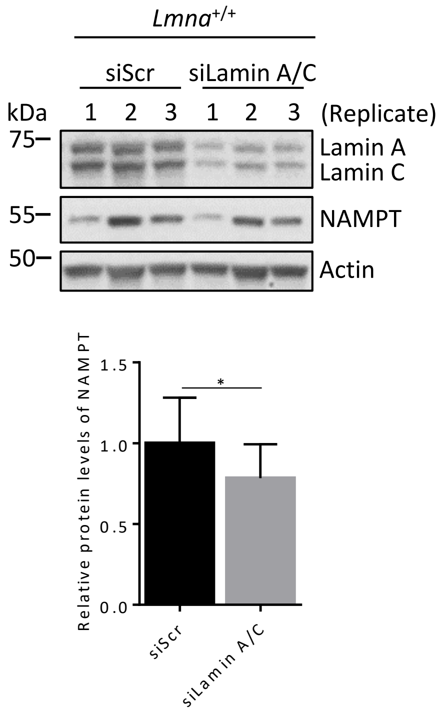

Supplementary Figure S3. The effect of siRNA knockdown in MEFs on the expression of the NAMPT enzyme. Western blotting of lysates from MEFs transfected with lamin A/C siRNA (siLamin A/C) and scrambled siRNA (siScr) revealed that NAMPT is reduced due to the lamin A/C knockdown, mirroring the data from *Lmna* knockout MEFs (*Lmna*$^{-/-}$ MEFs). The target sequence for siLamin A/C in mouse (Dharmacon ON TARGETplus, J-040758-05-0005) is 5′-GGAAGCAGCGAGAGUUUGA-3′. Data are presented as mean ± SD. Replicates refer to lysates prepared from three separate experiments. The P value was calculated using two-sided, paired Student's *t*-test; *P ≤ 0.05.

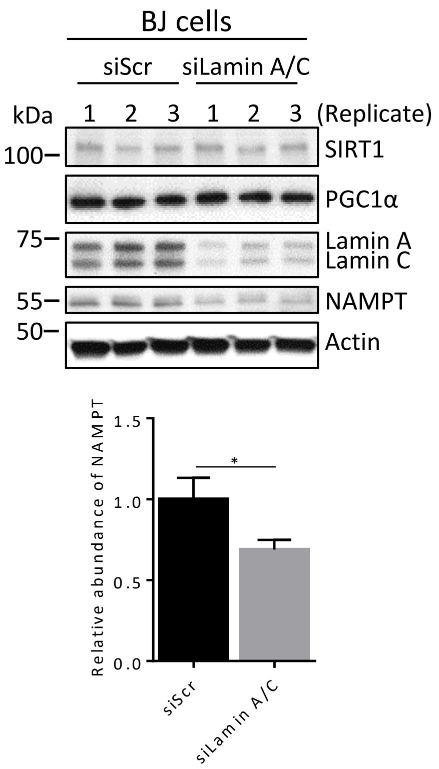

Supplementary Figure S4. The effect of siRNA knockdown in primary fibroblasts (BJ cells) on the expression of NAMPT, SIRT1, and PGC1α proteins. Western blotting of lysates from BJ cells transfected with lamin A/C siRNA (siLamin A/C) and scrambled siRNA (siScr) revealed that NAMPT is reduced due to the lamin A/C knockdown. The target sequence for siLamin A/C in humans (Dharmacon, J-004978-05) is 5′-GAAGGAGGGUGACCUGAUA-3′. Data are presented as mean ± SD. Replicates refer to lysates prepared from three separate experiments. The P value was calculated using two-sided, paired Student's *t*-test; *P ≤ 0.05.

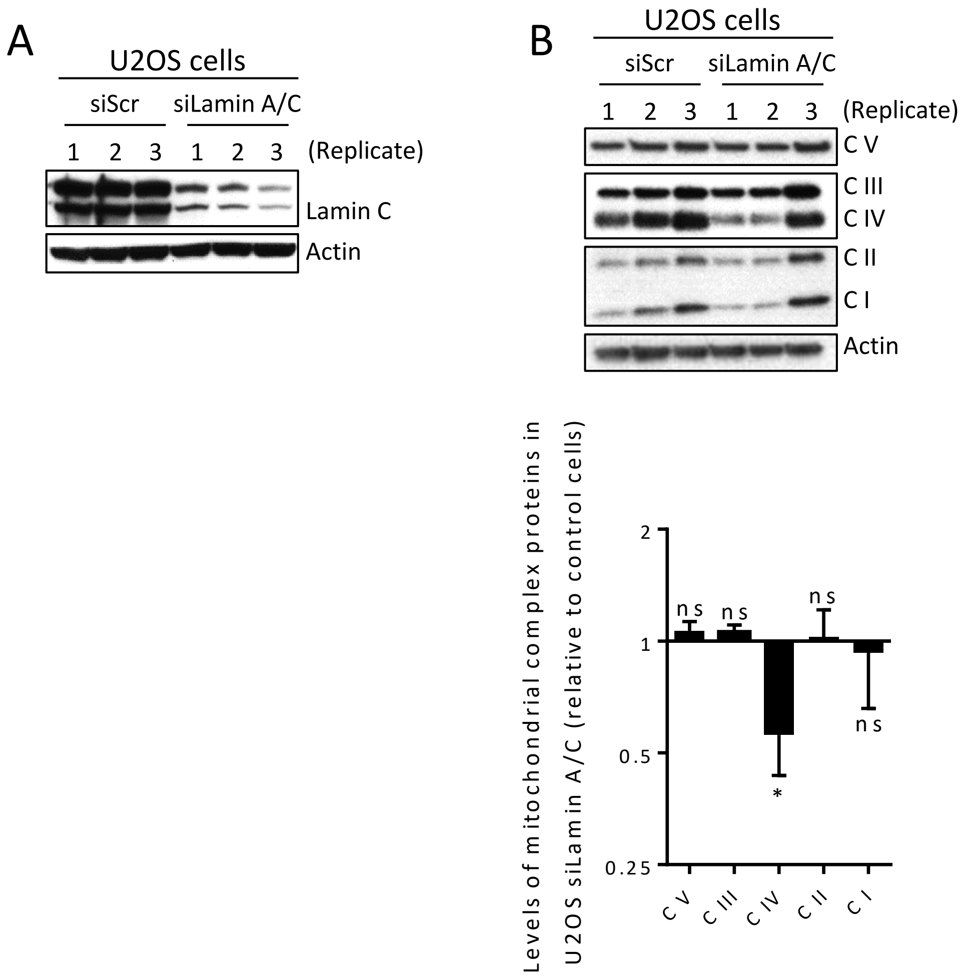

Supplementary Figure S5. Altered abundance of mitochondrial OXHPOS complexes due to lamin A/C depletion in U2OS cells. (A) siRNA knockdown of lamin A/C in U2OS cells. The target sequence for siLamin A/C in humans (Dharmacon ON TARGETplus, J-004978-05) is 5′-GAAGGAGGGUGACCUGAUA-3′. (B) Western blotting was performed on lysates from U2OS cells transfected with lamin A/C siRNA (siLamin A/C) or with scrambled siRNA (siScr), using total OXPHOS antibody cocktail (ab110413, Abcam) to determine the levels of specific subunits in the five OXPHOS complexes (CI-CV). Data are presented as mean ± SD. Replicates refer to lysates prepared from three separate experiments. All P values were calculated using two-sided, paired Student’s *t*-test; **P* ≤ 0.05; ns, non-significant. Identity of complexes: CI = NDUFB8 (NADH dehydrogenase [ubiquinone] 1 beta subcomplex subunit 8), CII = SDHB (Succinate dehydrogenase [ubiquinone] iron-sulfur subunit), CIII = UQCRC2 (Cytochrome b-c1 complex subunit 2), CIV = MTCO1 (mitochondrially encoded cytochrome c oxidase I), CV = vATP5A (ATP synthase, H + transporting, mitochondrial F1 complex, alpha 1).

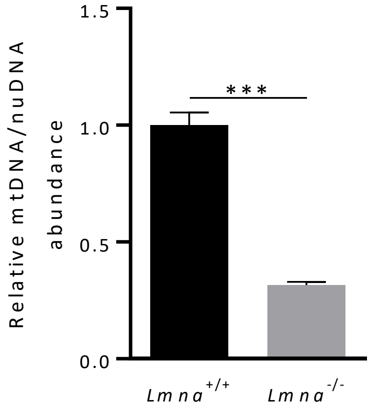

Supplementary Figure S6. Effect of *Lmna* deletion on mitochondrial DNA (mtDNA) abundance. The mtDNA and nuclear DNA (nuDNA) abundance was determined by using mtDNA and nuDNA PCR primers on total RNA samples isolated from *Lmna*$^{-/-}$ and *Lmna*$^{+/+}$ MEFs. The mtDNA abundance was normalized to nuclear DNA abundance. Data are presented as mean ± SD (n = 3). The P value was calculated using two-sided, unpaired Student's t-test; ***P ≤ 0.001.

A

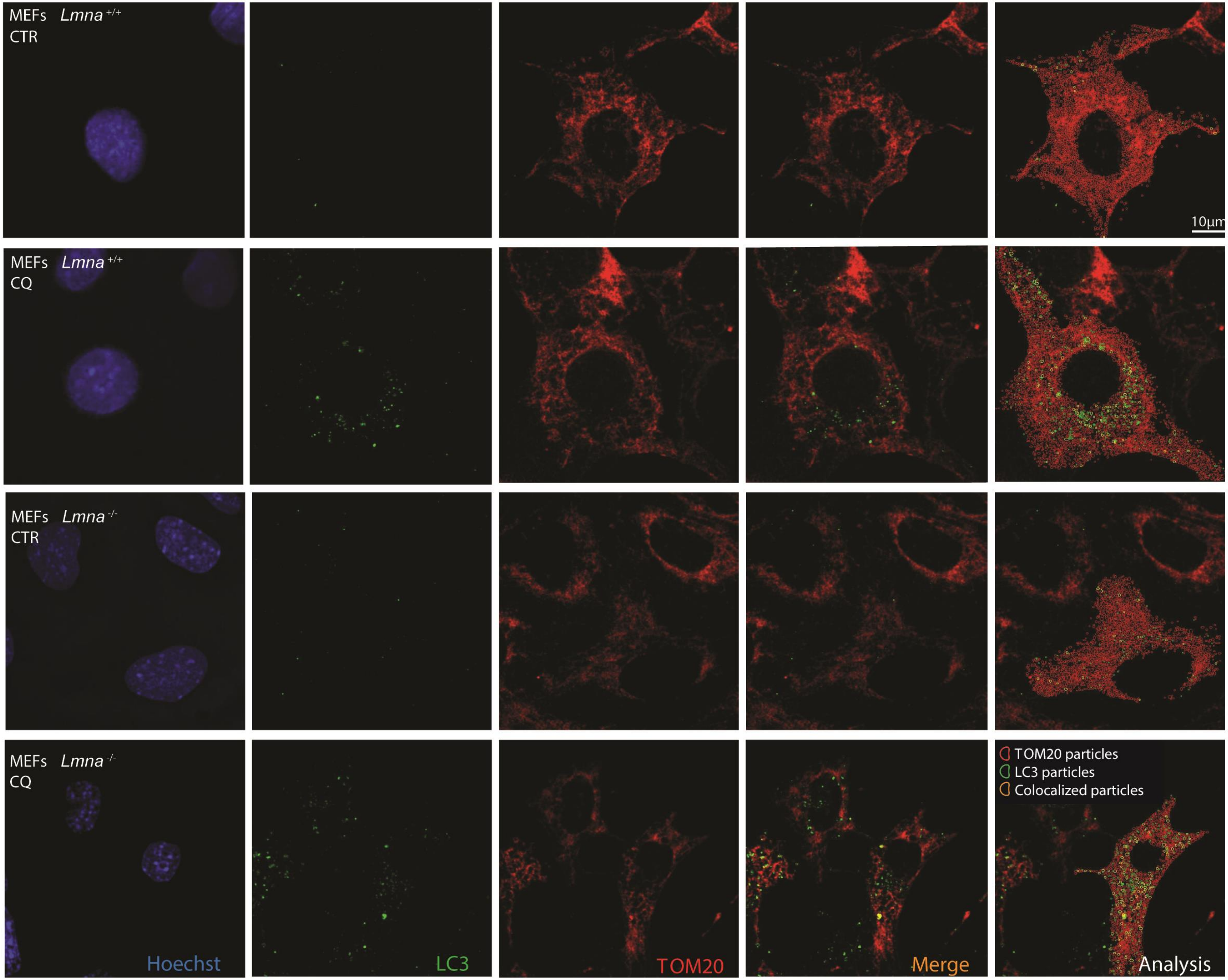
MEFs Lmna +/+
CTR
10µm
MEFs Lmna +/+
CQ
MEFs Lmna -/-
CTR
MEFs Lmna -/-
CQ
TOM20 particles
LC3 particles
Colocalized particles
Hoechst
LC3
TOM20
Merge
Analysis

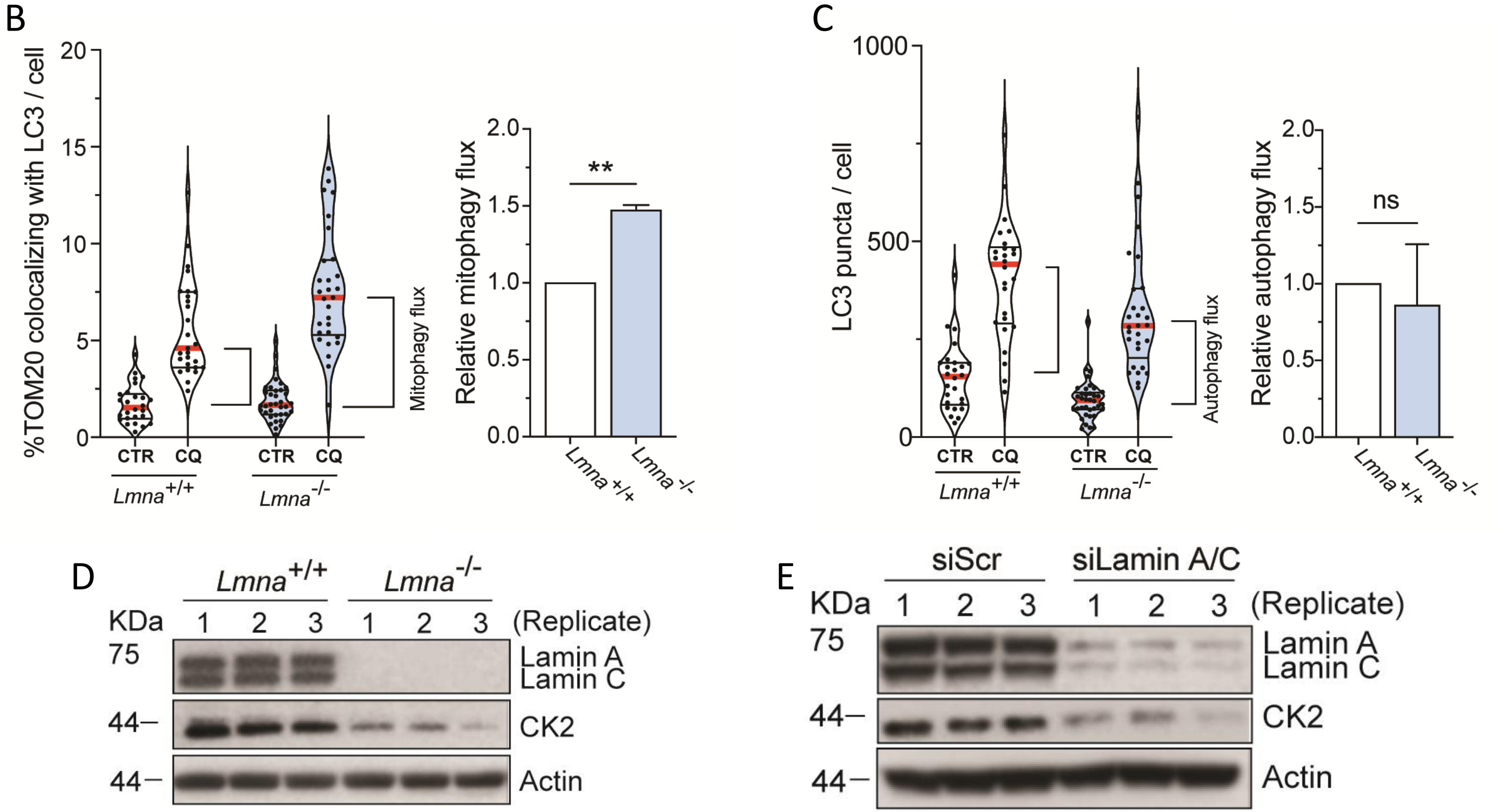

Supplementary Figure S7. Evidence for activation of mitophagy by lamin A/C depletion. (A-C) Basal mitophagy flux is enhanced in *Lmna*$^{-/-}$ MEFs. (A) Representative confocal fluorescence microscopy images of MEFs derived from wt (*Lmna*$^{+/+}$) or *Lmna*$^{-/-}$ mice upon incubation with 40 µM chloroquine (CLQ) for 5h to visualize mitochondria undergoing degradation through mitophagy. Anti-TOM20 (red) was used to visualize mitochondria; anti-LC3 (green) was used to identify autophagosomes. Percentage of mitochondria merging with LC3-positive puncta was calculated by Fiji analysis software. (B) Mitophagy flux determination. Values are expressed as % of mitochondria (TOM20+ particles) col-localizing with LC3/cell and graphed as violin boxes (25th-75th interquartile range) showing all points, with central bands representing the median of $n \geq 30$ different cells. Mitophagy rate was calculated by subtracting the % of TOM20 colocalizing with LC3 in control (CTR) (PBS-treated) cells from % of TOM20 colocalizing with LC3 in chloroquine (CQ)-treated cells. Results are shown as the mean ± SEM of 3 independent experiments. t test, ** $p < 0.01$. (C) Autophagy flux determination. Number LC3 puncta/cell graphed as violin boxes (25th-75th interquartile range) showing all points, with central bands representing the median of $n \geq 30$ different cells. Autophagy rate was calculated by subtracting the number of LC3 puncta of control (CTR) (PBS-treated) cells from number of LC3 puncta of CQ-treated cells. Results are shown as the mean ± SEM of 3 independent experiments. ns, not significant. (D-E) Lamin depletion results in reduction in mitochondrial inhibitor CK2. (D) lysates from *Lmna*$^{-/-}$ MEFs have lower CK2 than lysates from *Lmna*$^{+/+}$ MEFs. (E) Lysates from U2OS cells transfected with lamin A/C siRNA (siLamin A/C) have lower CK2 than lysates from scrambled siRNA (siScr). Replicates refer to lysates prepared from three separate experiments.

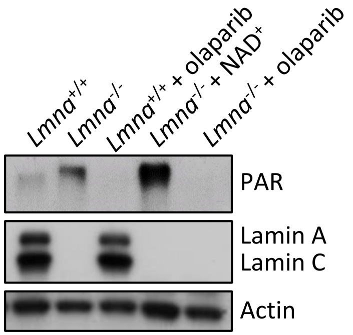

Supplementary Figure S8. Demonstration that olaparib inhibits PARylation in MEFs. Western blotting on lysates from *Lmna*$^{-/-}$ and *Lmna*$^{+/+}$ MEFs shows that olaparib treatment (2 μM for 2 h) is effective at inhibiting PAR formation. NAD^+ treatment (2 mM for 24 h) of *Lmna*$^{-/-}$ MEFs was included as a control (lane 4) to verify that the observed PAR bands represent PARP1 activation.

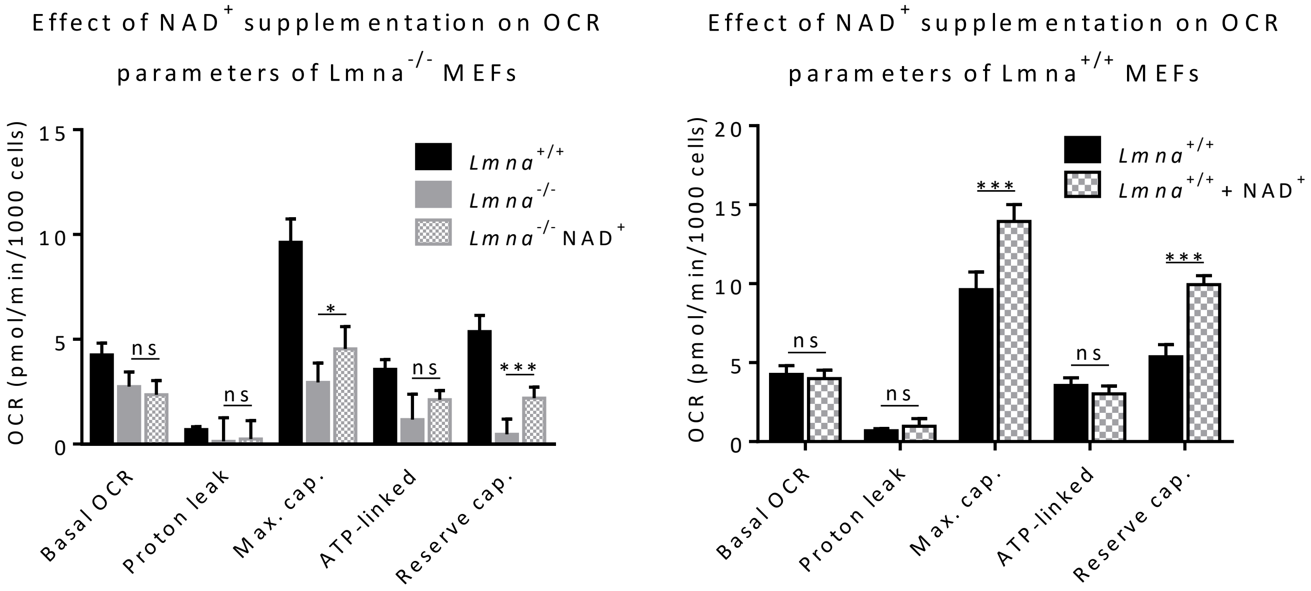

Supplementary Figure S9. NAD^+ supplementation (2mM NAD+ for 24 hours) results in significantly increased Maximum Capacity and Reserve Capacity in both *Lmna*$^{-/-}$ MEFs and Lmna+/+ MEFs, as detected using the Seahorse XF flux analyzer. Data are presented as mean ± SD (n = 4). All P values were calculated using two-sided, unpaired Student's *t*-test; ***P ≤ 0.001, *P ≤ 0.05; ns, non-significant. cap., capacity.

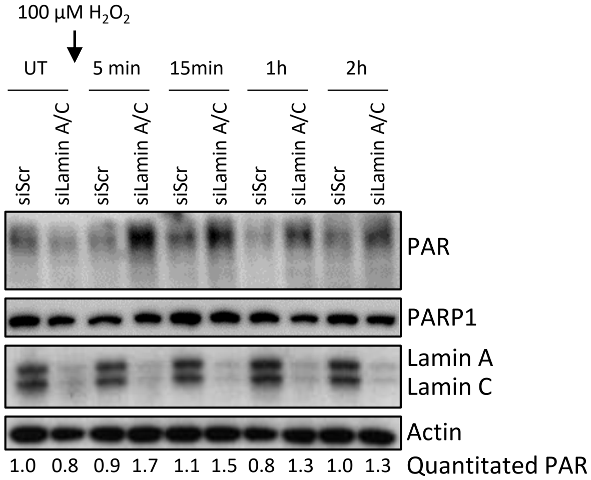

Supplementary Figure S10. The effects of oxidative stress on PARylation in a lamin A/C-depleted cellular environment. Western blotting on lysates from U2OS cells transfected with lamin A/C siRNA (siLamin A/C) or with scrambled siRNA (siScr) shows that the siLamin A/C cells have enhanced PAR formation (relative to siScr U2OS) during the indicated times after a 1h H_2O_2 treatment at 100 μM. The relative abundance of PAR corrected for actin is written below the image. PARP1 protein levels verify that the observed increases in PAR levels are not due to any increases in PARP1 levels. The target sequence for human lamin A/C (Dharmacon, J-004978-05) is 5′-GAAGGAGGGUGACCUGAUA-3′.

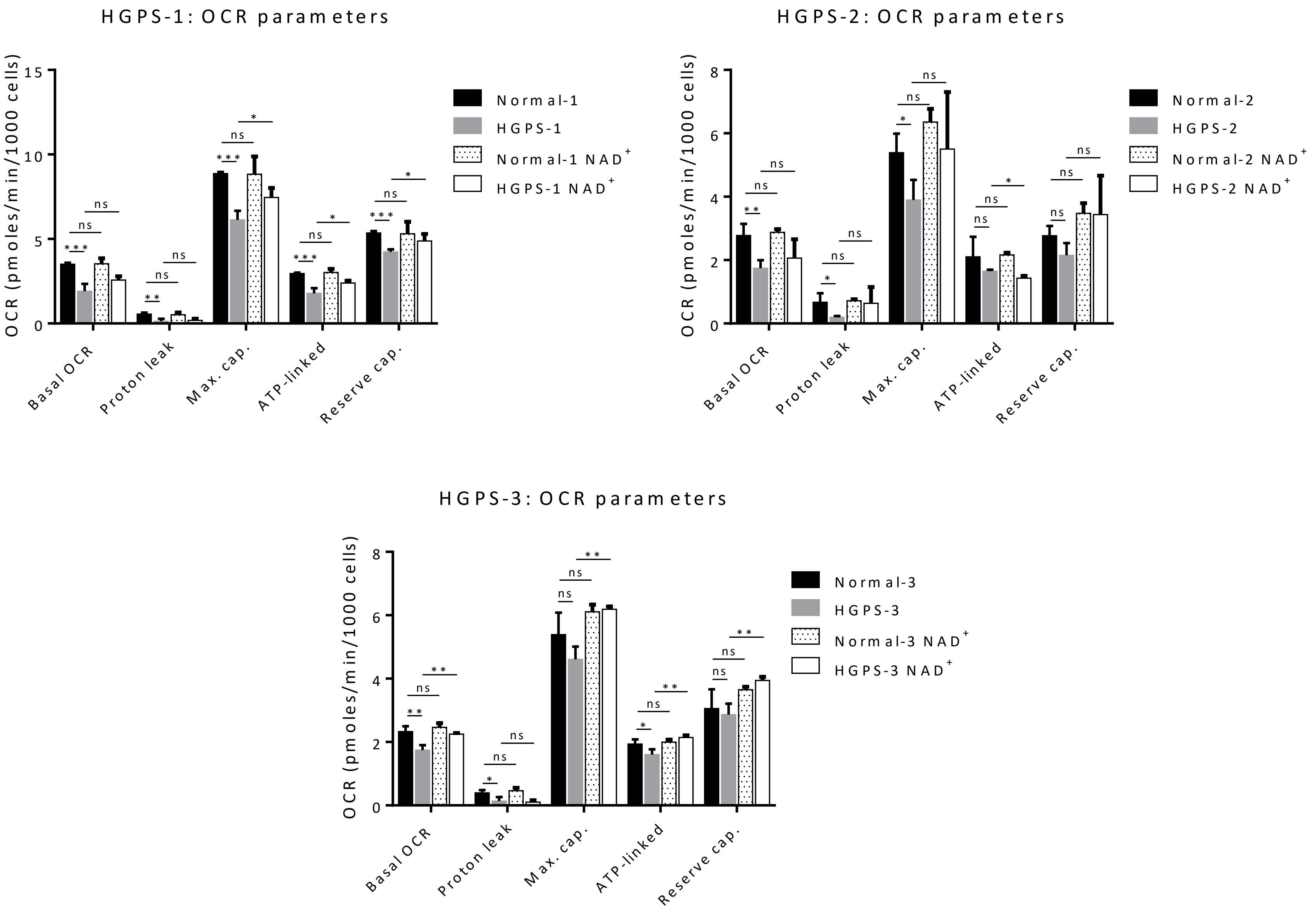

Supplementary Figure S11. The effect of NAD+ supplementation (2mM NAD+ for 24 hours), on bioenergetic parameters in HGPS fibroblasts and corresponding normal cells, as detected using the Seahorse XF flux analyzer. Data are presented as sample mean ± SD (n = 4). All P values were calculated using two-sided, unpaired Student's *t*-test; ***P ≤ 0.001, **P ≤ 0.01, *P ≤ 0.05; ns, non-significant. cap., capacity.

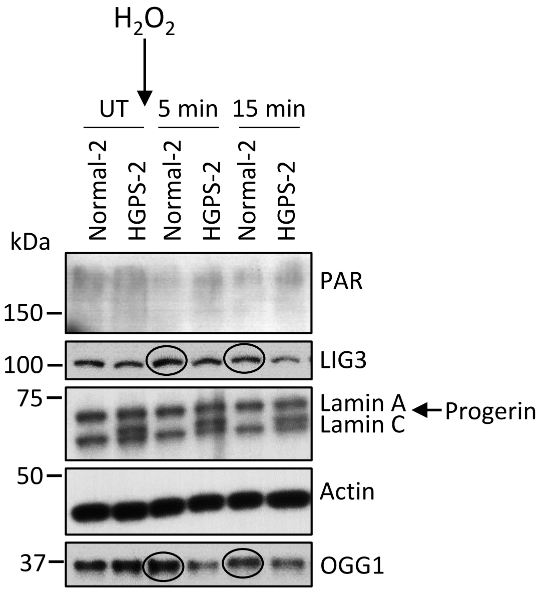

Supplementary Figure S12. Enhanced LIG3 and OGG1 (circled) in normal fibroblasts compared to HGPS fibroblasts after oxidative stress may contribute to enhanced BER, less DNA damage and thus less PAR formation in the normal cells relative to HGPS cells. PAR formation was examined, by immunoblotting, before and after oxidative stress (100 µM H2O2 for 1h) in HGPS-2 and Normal-2 fibroblasts. Progerin can be seen as a band between the lamin A and C isoforms.
